# Effects of traditional Chinese exercises or their integration with medical treatments on cognitive impairment: a network meta-analysis based on randomized controlled trials

**DOI:** 10.3389/fnagi.2024.1475406

**Published:** 2024-11-11

**Authors:** Jiadong Qiu, Sungmin Kim

**Affiliations:** ^1^Department of Sports Science, Hanyang University, Seoul, Republic of Korea; ^2^BK21 FOUR Human-Tech Convergence Program, Hanyang University, Seoul, Republic of Korea; ^3^Center for Artificial Intelligence Muscle, Hanyang University, Seoul, Republic of Korea

**Keywords:** Tai Chi, traditional Chinese exercises, Qigong, cognitive impairment, medical treatments

## Abstract

**Objective:**

This study aims to summarize and critically evaluate the effects of traditional Chinese exercises, both in isolation and in combination with medical treatments, on cognitive impairment.

**Methods:**

A systematic search of academic databases, including PubMed, Embase, Web of Science, Cochrane Library, CNKI, Wanfang, and VIP, was conducted to identify the randomized controlled trials (RCTs) that evaluated traditional Chinese exercises and their integration with medical treatments for addressing cognitive impairment. Study quality was assessed using the Cochrane Handbook’s Risk of Bias tool. A total of 24 RCTs involving 1,808 participants were included. The primary outcome measures were the Montreal Cognitive Assessment (MOCA) and the Mini-Mental State Examination (MMSE). Subgroup analyses were performed to compare the intervention effects.

**Results:**

The network meta-analysis revealed that acupuncture combined with Tai Chi (Aandtaiji) showed the most significant improvement in MOCA scores, followed by Qigong. Tai Chi soft ball exercise (Taijiball) demonstrated the greatest improvement in MMSE scores.

**Conclusion:**

The combination of traditional Chinese exercises with medical treatment is more effective in improving MOCA scores, while traditional exercises alone yield better results to enhance MMSE scores. The extended practice of Tai Chi and Qigong enhances cognitive function in patients with cognitive impairment.

## Introduction

As the aging continues to accelerate, cognitive impairment has become a widespread and urgent public health issue, affecting millions of people worldwide (Loss et al., 2024; Moawad et al., 2024). Cognitive impairment refers to a decline in abilities like information processing, attention, memory, language, and executive functions, which significantly impact daily life (Arnold, 2020). Cognitive impairment is an umbrella term that encompasses a range of conditions, from mild cognitive impairment (MCI) to severe dementia (Sohn, 2018). Across the world, the care and treatment costs for Alzheimer’s and related dementias are estimated at $1.3 trillion annually, covering medical, home care, long-term facilities, and caregiver services (Roth, 2022; Pandharipande et al., 2023). Low-cost treatment and healthcare strategies are urgently needed to reduce the financial strain on patients and families, slow cognitive decline, and lessen the demand for professional care and healthcare resources (Lin et al., 2024).

Traditional Chinese exercises, such as Qigong, Tai Chi, and Taijiball, combine breath control, body postures, and meditation to enhance physical and mental health (Li et al., 2001; Park et al., 2023; Duan et al., 2024). These exercises have proven effective in aiding recovery from chronic diseases, including cognitive impairment, and in reducing treatment costs (Wayne et al., 2014; Li et al., 2019; Yao et al., 2023). Traditional Chinese exercises combined with medical interventions are considered potential effective solutions due to their low cost, ease of implementation, and long-term efficacy (Navas-Otero et al., 2024; Zhang X. et al., 2024).

However, previous meta-analyses have limitations. Most studies focus mainly on Yang-style Tai Chi and a few types of Qigong (Park et al., 2023; Wang et al., 2023), such as Baduanjin Qigong (BDJqigong), Yi Jin Jing Qigong, Wuqinxi Qigong, and Liuzijue Qigong (LZJqigong), have been considered (Yao et al., 2023). Overlooking other traditional exercises and the diversity within Tai Chi and Qigong. Tai Chi includes various styles, such as Chen, Yang (including simplified forms like the 24-style; Lei et al., 2022), six-style (Lin et al., 2019), and eight-style (Meiling, 2021), Wu style, Sun style, and He style (You et al., 2021). Many commonly practiced Qigong forms, such as SEDJqigong (Jing et al., 2019; Hengjia et al., 2020), Yangfeifang Qigong (YFF Qigong) (Min and Xiaodan, 2023), and exercises like Taijiball (Yong et al., 2016), have never been included in meta-analyses.

Furthermore, combinations of traditional Chinese exercises with therapies like acupuncture and transcranial direct current stimulation (tDCS) are rarely included (Rizhen et al., 2013; Yu, 2021; Liu et al., 2022; Xu et al., 2023). However, these combinations are seldom included in the meta-analyses, and these limitations restrict the generalizability of findings. Moreover, meta-analyses have provided mixed results. Lyu et al. (2021) found Tai Chi had no significant effect on cognitive dysfunction, while Zhou et al. (2022) reported significant improvements.

Most previous studies relied on traditional meta-analysis, limited to direct comparisons between the two treatments (Lyu et al., 2021). Unlike traditional meta-analysis, network meta-analysis allows for the simultaneous comparison of multiple treatments, including traditional Chinese exercises and their combination with medical treatments, even when direct comparisons are unavailable. It ranks interventions by effectiveness, offering a more comprehensive understanding of therapeutic potential. This method is particularly useful in medical research with diverse treatment options, as it integrates both direct and indirect evidence, enhances statistical power, and reveals benefits not apparent in direct comparisons, making it a valuable tool for clinical decision-making (Mbuagbaw et al., 2017; Tian and Li, 2024).

This network meta-analysis review aims to assess the impact of various traditional Chinese exercises, alone and combined with other treatments, on cognitive function in individuals with cognitive impairment. The goal is to identify the most effective intervention strategies, providing valuable insights for clinical trials.

## Methods

### Study registration

The study was conducted in accordance with the Preferred Reporting Items for Systematic Reviews and Meta-Analyses for Network Meta-Analyses (PRISMA-NMA) guidelines and was prospectively registered with PROSPERO (CRD42024536140).

## Eligibility criteria

### Inclusion criteria

The inclusion criteria were formulated based on the PICOS (population, interventions, controls, outcomes, study design) framework.

*Population:* Patients with a confirmed diagnosis of cognitive impairment, who are in the early to early-middle stages of dementia or mild cognitive impairment (MCI), with no prior use of any medications before the intervention were included in the analysis. There were no restrictions on age, gender, race, or nationality.

*Intervention:* Studies focusing on traditional Chinese exercises (such as Tai Chi, Qigong, etc.) combined with other therapeutic methods (such as acupuncture, tDCS, etc.) were included for the analysis. These interventions were applied either individually or in combination.

*Comparison*: The control group in this study comprised participants who maintained their regular daily activities without receiving any form of intervention.

*Outcome:* Cognitive screening and assessment are crucial for evaluating cognitive impairment. In epidemiological studies, MOCA and MMSE are commonly used tools. Thus, the results of this study are based on these measures (Yu et al., 2012; Arevalo-Rodriguez et al., 2021; Jia et al., 2021).

*Main outcome measure*: Based on the results of this study, the Aandtaiji demonstrated the best effect on the MOCA scores, while Taijiball showed the most significant improvement of the MMSE scores.

*Second primary outcome measure*: Based on the results of this study, the intervention combining traditional Chinese exercises with other therapeutic methods was superior to all other traditional Chinese exercises in the MOCA scores when compared to the control group receiving no treatment. However, traditional Chinese exercise interventions alone were more effective in improving the MMSE scores than the integrated approach of traditional exercises with other therapeutic methods.

*Study design*: The study design was restricted to randomized controlled trial.

### Exclusion criteria

Studies involving intervention methods unrelated to traditional Chinese exercises in the experimental group; duplicate studies or data, animal experiments, non-patient studies, studies not published in Chinese or English, and studies unrelated to cognitive impairment were excluded from the analysis.

## Data sources and search strategy

We conducted a systematic search across major academic databases, including PubMed, EMbase, Web of Science, Cochrane Library, CNKI, Wanfang, and VIP, up to April 30, 2024. The aim was to identify randomized controlled trials (RCTs) evaluating the effects of traditional Chinese exercises (e.g., Tai Chi) or their combination with other treatments on cognitive impairment. Both subject headings and free-text terms were used to capture the relevant studies. To minimize bias and errors, the search strategy followed these principles: (1) *diverse search terms*: We included a broad range of synonyms and variants, such as Tai Ji, TAIJI, Taijiquan, Baduanjin, cognitive impairment, mild cognitive impairment, dementia, and others; (2) *language restrictions*: The search was limited to English and Chinese publications to include global research within these two languages; (3) *manual and gray literature search*: Additional studies were identified by manually reviewing reference lists and including gray literature (e.g., dissertations). This strategy ensures a comprehensive collection of studies on traditional Chinese exercises and their effects on cognitive impairment, minimizing bias and enhancing the reliability of the findings.

### Study selection

Based on the predetermined literature retrieval strategy, researchers Jiadong Qiu and Wanyu Shu utilized the EndNote X20 software to retrieve and eliminate duplicate documents. The remaining literature has been assessed for inclusion or exclusion based on established criteria through title and abstract review, followed by a thorough evaluation of the full texts for qualification. In cases of discrepancy, a third researcher, Liang Chen, was consulted to reach a consensus on the final selection of literature.

### Data extraction

Two researchers, Jiadong Qiu and Wanyu Shu, developed a data extraction form based on the necessary information for the study and conducted independent data extraction. The included contents were as follows: (1) basic information such as title, author, year, study type, diagnostic criteria, intervention measures, treatment course, and outcome indicators; (2) demographic characteristics including sample size, age, and gender; and (3) methodology of information: Various methods such as randomization, allocation concealment, and blinding are employed in research studies. In cases where discrepancies arise in the data collected by two individuals, they engaged in a discussion to resolve the issue. If a resolution cannot be reached, further discussion and decision-making processes were undertaken.

### Risk of bias in studies

Two researchers Jiadong Qiu and Wanyu Shu used the Cochrane Risk of Bias Assessment tool for RCTs to assess the risk of bias. The assessment tool contains the following seven items: generation of the random sequence, allocation concealment, blinding of subjects and intervention providers, blinding of outcome assessors, incomplete outcome data, selective outcome reporting, and other sources of bias, with each item assessing the outcome as low bias, high bias, or unclear (Isenberg et al., 2020).

### Synthesis methods

Bayesian random effects models were employed to assess the comparative effectiveness of different interventions. The Markov chain Monte Carlo method was utilized for modeling, with four Markov chains run concurrently and annealing performed 20,000 times. Model convergence was achieved after 50,000 simulation iterations. The study utilized the bias information criterion (BIC) to assess the model fit and global consistency, employed the node splitting method to evaluate local consistency in the presence of closed-loop networks, ranked interventions according to the surface under the cumulative ranking (SUCRA) curve, and generated a league table to compare the efficacy of various interventions. We extracted data from the original 24 RCTs. Numerous original studies incorporated in the analysis conducted multi-arm studies on Tai Chi and other traditional Chinese exercises within the MOCA and MMSE indexes, categorizing subgroups based on specific exercise types. Utilizing Bayesian network meta-regression, we examined potential differences between various traditional Chinese exercises across different intervention durations compared to a control group. When the number of studies included in the outcome index exceeded 10, a funnel plot was utilized to visually represent publication bias. Statistical analyses were conducted using Stata 15.0 (Stata Corporation, College Station, TX) and R 4.2.0 (R Development Core Team, Vienna).^1^ A significance level of *p* < 0.05 indicated a statistically significant difference.

## Results

### Study selection

**Figure 1:**
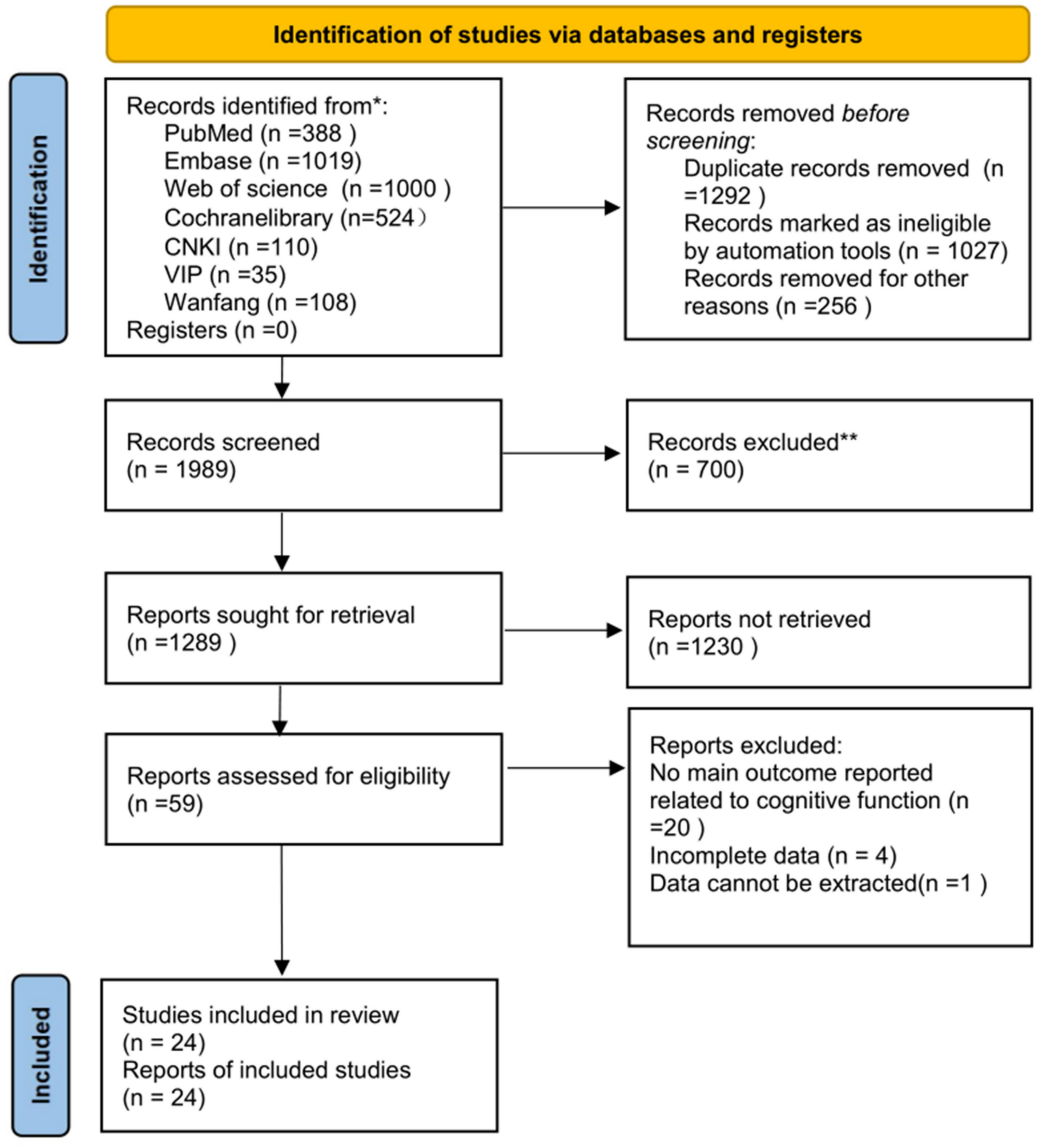
Literature screening process.

The literature search and study selection process yielded a total of 3,281 records from the database (Supplementary Table 1). Through screening the references and related articles of the included studies, 1,027 duplicate articles were excluded using software, and an additional 256 duplicate articles were removed by comparing titles with authors. In total, 1,292 duplicate articles were eliminated, resulting in a final selection of 1989 articles. Following the initial screening of titles and abstracts, 700 articles were excluded, leaving a total of 1,289 articles for further review. Of these, 59 underwent full-text evaluation, resulting in the inclusion of 24 studies in the final analysis (Figure 1).

### Study characteristics

This study included 24 studies (Burgener et al., 2008; Rizhen et al., 2013; Tuan and Juan, 2013; Cheng et al., 2014; Li et al., 2014; Chan et al., 2016; Yong et al., 2016; Jun and Zhongxing, 2018; Siu and Lee, 2018; tao et al., 2018; Jing et al., 2019; Lin et al., 2019; Hengjia et al., 2020; Liu et al., 2021; Meiling, 2021; Yu, 2021; Zhicheng et al., 2021; Jinping, 2022; Liu et al., 2022; Yu et al., 2022; Chen et al., 2023; Min and Xiaodan, 2023; Xia et al., 2023; Xu et al., 2023) with a total of 1808 patients. The authors were from China and the United States, and the publication time was from 2008 to 2023. Most of the studies were published in the past 5 years. The criteria of MCI mainly came from Petersen. Twenty-four studies employed the same type of traditional Chinese exercises or combined them with other medical interventions (Table 1).

**Table 1:** Characteristics of included studies in this review.

No	Title	Language	Author	Country	Time	Patient	Intervention	Number	Course	Outcome
1	Tai Ji Quan and global cognitive function in older adults with cognitive impairment: A pilot study	English	Fuzhong Li	USA and China	2014	MCI	14-week Yang style tai Ji: Moving for Better Balance (TJQMBB) program;	Taiji group (*n* = 20) Control group (*n* = 20)	14w	MMSE
2	Tai Chi versus conventional exercise to improve cognitive performance in older adults with mild cognitive impairment	English	Angus P Yu	Hong Kong, China	2022	MCI	Tai Chi group: 24 weeks of Yang-style Tai Chi training three times a week for 60 min each session; Conventional Exercise group: 24 weeks of fitness training three times a week for 60 min each session; Control group: no intervention.	Tai Chi group (*n* = 10); Exercise group (*n* = 12); Control group (*n* = 0.12)	24w	MOCA
3	Tai chi qigong as a means to improve night-time sleep quality among older adults with cognitive impairment: A pilot randomized controlled trial	English	Aileen WK Chan	Hong Kong, China	2016	MCI	Yang and Wu style tai Ji and Baduanjin, Wuqinxi, Liuzijue qigong sessions twice a week for 2 months, 60 min each session	Taiji group (*n* = 27) Control group (*n* = 25)	8w	MMSE
4	Simplified Tai Chi 6-Form Apparatus for Balance in Elderly People with Alzheimer’s Disease	English	Lin, Y. C.	Taiwan, China	2020	MCI	The Yang style tai Ji group (TCGr) completed an 8-week training course for the Simplified Yang style tai Ji 6-Form Apparatus	Taiji group (*n* = 11) Control group (*n* = 10)	8w	MMSE
5	Mind–Body Exercise Modulates Locus Coeruleus and Ventral Tegmental Area Functional Connectivity in Individuals With Mild Cognitive Impairment	English	Liu Jiao	China	2021	MCI	Participants were randomized into Qigong, brisk walking, or a healthy education control group for 6 months. The exercise groups participated in sessions 3 days/week, 60 min/day.	Qigong group (*n* = 20) Control group (*n* = 20)	24w	MOCA
6	Mental and Physical Activities Delay Cognitive Decline in Older Persons with Dementi	English	Jing Tao;	Hong Kong China	2014	Early to early-middle stages of dementia	Mahjong. Taiji group. Control group	Mahjong (*n* = 36) Taiji group (*n* = 39) Control group (*n* = 35)	24w	MOCA
7	Effects of Tai Chi on cognition and instrumental activities of daily living in community dwelling older people with mild cognitive impairment	English	Siu, Mei Yi	Hong Kong China	2017	MCI	The intervention group received 16 weeks of Yang-style Tai Chi training, two sessions per week, each lasting 1 h	Taiji group (*n* = 80) Control group (*n* = 80)	16w	MMSE
8	Effects of Tai Chi combined with tDCS on cognitive function in patients with MCI: a randomized controlled trial	English	Ying Xu	USA and China	2023	MCI	Taiji and tDCS.Tai Ji. ontrol	Taiji and tDCS (*n* = 44) Tai Ji (*n* = 49) ontrol group (*n* = 44)	24w	MOCA
9	Effects of Tai Chi Chuan on Cognitive Function in Adults 60 Years or Older With Type 2 Diabetes and Mild Cognitive Impairment in China: A Randomized Clinical Trial	English	Yannan Chen	USA and China	2023	MCI	Yang style tai Ji and walking training, both for 60 min/session, 3 times/wk. for 24 weeks	Taiji group (*n* = 110) Control group (*n* = 110)	36w	MOCA
10	Effects of mind–body exercise baduanjin on cognition in community-dwelling older people with mild cognitive impairment: a randomized controlled trial	English	Xia Rui	China	2022	MCI	Baduanjin exercise group received 24 weeks of Baduanjin exercise training, 60 min sessions, 3 days per week; Brisk walking group received 24 weeks of brisk walking, 60 min per session, 3 sessions per week.	Qigong group (*n* = 70) Control group (*n* = 65)	24w	MOCA
11	Effects of exergaming based Tai Chi on Cognition and dual task gait in older adults with mild cognitive Impairment a randomized control trial	English	Chen Liang Liu	China	2022	MCI	EXER-TC and TC groups received 36 training sessions (three 50-min sessions per week) for 12 weeks	EXER-taiji (*n* = 16) Taiji group (*n* = 17) Control group (*n* = 19)	12 W	MOCA
12	The effects of a multimodal intervention on outcomes of persons with early-stage dementia	English	Sandy C. Burgener	USA	2008	Early to early-middle stages of dementia	40-week intervention, including Taiji exercises, and support group participation	Taiji group (*n* = 24) Control group (*n* = 19)	40w	MMSE
13	Clinical study of acupuncture combined with Tai Chi in the treatment of mild cognitive impairment caused by cerebral small vessel disease	Chinese	Ze Yu Shen	China	2021	MCI	Acupuncture combined with Yang style tai Ji intervention; the control group used only Yang style tai Ji intervention.	A + Taiji (*n* = 31); Control group (*n* = 31)	8w.	MOCA
14	A study on the evaluation of the clinical efficacy of Baduanjin in patients with mild cognitive dysfunction	Chinese	Qian Yang	China	2019	MCI	Ba duan jin qigong 5 times a week, 40 min each session, for 24 weeks. The control group received no intervention	Qigong group (*n* = 32) Control group (*n* = 32)	24w	MOCA
15	The effect of Naoling decoction combined with Tai Chi on the rehabilitation of patients with Alzheimer’s disease	Chinese	Ri Zhen Li	China	2013	Early-stage Alzheimer’s disease	he treatment group received Naoling Decoction combined with Yang style tai Ji exercise, while the control group received conventional treatment	Naoling +Ytaiji (*n* = 32) Control group (*n* = 30)	12w	MMSE
16	Study on the Rehabilitation Effects of Six-Character Formula of Health Qigong on Mild Cognitive Impairment in the Elderly	Chinese	Xin Tuan Zheng	China	2013	MCI	“Six Healing Sounds” fitness Qigong exercise, practiced twice daily for 30 min each session, 5 days a week for 6 months	Qigong group (*n* = 45) Control group (*n* = 43)	24w	MOCA
17	The Impact of Virtual Reality-Based Baduanjin Exercise on Mild Cognitive Impairment in Elderly Patients in Nursing Homes	Chinese	Sun Zhi Chen	China	2021	MCI	VR-based qigong 50 min per session, three times a week for 24 weeks	VR qigong (*n* = 29) Control group (*n* = 28)	24w	MOCA
18	Study on the Intervention Effect of Continuous Health Qigong Exercise on Mild Cognitive Impairment in the Elderly	Chinese	Jun Cai	China	2018	MCI	6 months of fitness Qigong exercises including Yi Jin Jing, Ba Duan Jin, Wu Qin Xi, Liu Zi Jue	Qigong group (*n* = 28), Control group (*n* = 30)	24w	MOCA, MMSE
19	Clinical Observation of the Efficacy of Eight-Style Tai Chi on Mild Cognitive Impairment Caused by Lacunar Infarction	Chinese	Mei Ling Huang	China	2021	MCI	Yang style tai Ji group practiced Eight Style Taijiquan for 30 min	Taiji group (*n* = 33) Control group (*n* = 33)	24w	MOCA
20	Clinical Study of Baduanjin Combined with Transcranial Direct Current Stimulation in Treating Mild Cognitive Impairment After Stroke	Chinese	Jin Ping Feng	China	2022	MCI	The control group received transcranial direct current stimulation, while the observation group Baduanjin exercise	T + qigong (*n* = 47) Control group (*n* = 47)	8w	MOCA,MMSE
21	The Impact of Baduanjin on the Cognitive Levels of Patients with Mild Cognitive Impairment	Chinese	Tao Liu	China	2018	MCI	Baduanjin exercise intervention for 6 months	Qigong group (*n* = 30) Control group (*n* = 30)	24w	MOCA
22	Experimental Study on Tai ji Soft Ball Exercise in the Treatment of Senile Dementia	Chinese	Yong Zhou	China	2016	Early to early-middle stages of dementia	Taijiball(softball) exercise under professional guidance, compared to jogging in the control group	Taijiball group (*n* = 18) Control group (*n* = 18)	32w	MMSE
23	Study on the Impact and Mechanism of Health Qigong Yangfei Prescription on Cognitive Function in Patients with Stable Chronic Obstructive Pulmonary Disease	Chinese	Min Zhuang	China	2023	Severity levels GOLD 1–3 (FEV1 ≥ 30%)	Yangfeifang Qigong exercise, 5 days a week, 2 sessions per day, 35 min per session for 12 weeks	Qigong group (*n* = 18) Control group (*n* = 18)	12w	MOCA
24	59.Study on the Impact of Health Qigong 12 Duanjin on Patients with Mild Cognitive Impairment	Chinese	Heng Jia Liu	China	2020	MCI	Twelve Dan Jin qigong Exercises performed 5 times per week, 40 min per session, over 24 weeks	Qigong group (*n* = 30) Control group (*n* = 30)	24w	MOCA

### Risk of bias in studies

**Figure 2:**
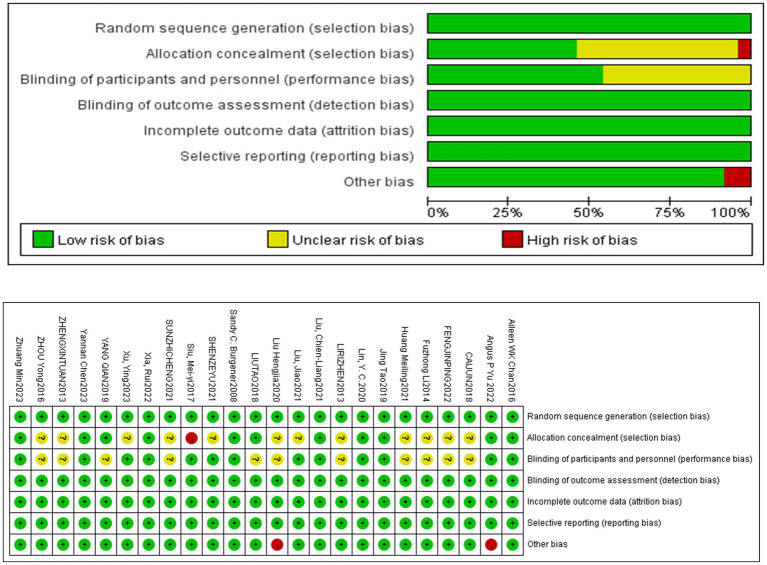
Summary of risk of bias for all the included studies. Method: this figure presents a risk of bias assessment based on the Cochrane Collaboration’s framework. Bias categories include random sequence generation, allocation concealment, blinding, and other potential biases. Green: Low risk of bias. Yellow: Unclear risk of bias. Red: High risk of bias. Results: The bar chart summarizes the proportion of studies with low, unclear, or high risk of bias. Most categories show a low risk, with some uncertainty in allocation concealment and a small portion of high risk in “other bias.” A table below shows individual study assessments, with color-coded dots indicating the bias level.

This study reviewed 24 articles. Regarding randomization, all studies provided detailed descriptions or explicitly stated adherence to random allocation procedures, resulting in a “low-risk” classification. For allocation concealment, one study explicitly reported not implementing it (Siu and Lee, 2018), 11 studies confirmed its use, while 12 studies lacked sufficient detail and were classified as having an “uncertain risk of bias.” Due to the nature of the interventions, blinding the participants was limited. Thirteen studies reported using blinding, while the others were rated as having “uncertain risk” due to inadequate reporting. For outcome assessment, data completeness, and selective reporting, all studies were deemed “low risk” as participant numbers were consistent with initial enrollments, and no selective reporting was observed. Two studies with small sample sizes (Hengjia et al., 2020; Yu et al., 2022) raised concerns about publication bias and were rated as high risk. All other studies were assessed as low risk. Figure 2 summarizes the overall risk of bias and provides individual assessments for each study (Figure 2).

### Meta-analysis

The first outcome measure was MOCA linkages between interventions. MOCA: 16 studies (Tuan and Juan, 2013; Jun and Zhongxing, 2018; tao et al., 2018; Jing et al., 2019; Hengjia et al., 2020; Liu et al., 2021; Meiling, 2021; Yu, 2021; Zhicheng et al., 2021; Jinping, 2022; Liu et al., 2022; Yu et al., 2022; Chen et al., 2023; Min and Xiaodan, 2023; Xia et al., 2023; Xu et al., 2023) used MOCA as an outcome indicator and four studies (Yu, 2021; Jinping, 2022; Liu et al., 2022; Xu et al., 2023) used a combination of traditional Chinese exercises and medical means, among which the direct comparison between Qigong and control group was the most, followed by the direct comparison between Taiji (Tai Chi) and control group, and the combination of Taiji and other interventions was the most. The figure shows a closed loop (Supplementary Figure 1).

### Synthesized results

**Figure 3:**
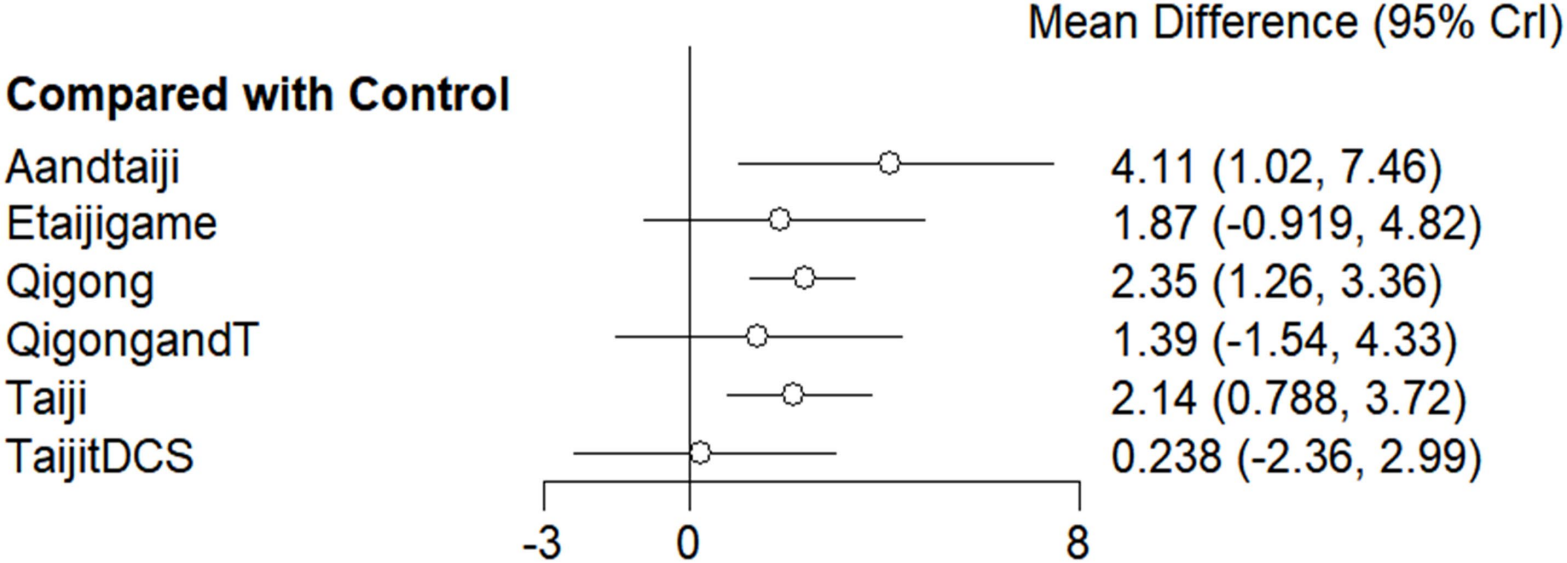
Comparison of MOCA index intervention and control groups (Forest Plot). 1: Qigong; 2: Qigong and Transcranial Direct Current Stimulation (tDCS); 3: Exergaming-based Tai Ji (body movement-controlled computer Tai Ji game); 4: Tai Ji; 5: Acupuncture and Tai Ji (Aandtaiji); 6: Tai Ji combined with tDCS (TaijitDcs). Method: This forest plot shows the mean differences between various interventions and the control group, with 95% credible intervals (CrI). Circles represent point estimates, and horizontal lines indicate credible intervals. Results: Significant positive effects were observed for interventions like Acupuncture and Tai Ji (Aandtaiji), followed by Qigong. Other interventions, such as Exergaming and Qigong with tDCS, showed unclear effects.

As regards MOCA indicators, the network meta-analysis results showed that compared with the control group, the three interventions (Aandtaiji, Qigong, and Taiji) showed significant advantages (*p* < 0.05) (Figure 3). The top three interventions for MOCA indicators were Aandtaiji (Acupuncture combined with Tai Chi) [RR = 4.11, 95% CI (1.102, 7.46), *p* < 0.05], Qigong [RR = 2.35, 95% CI (1.26, 3.36), *p* < 0.05], and Taiji [RR = 2.14, 95% CI (0.79, 3.72), *p* < 0.05], all showing significant effects (Supplementary Table 2). However, although Taiji-tDCS (Tai Chi combined with tDCS) [RR = 0.24, 95% CI (−2.36, 2.99), *p* < 0.05] was included in the analysis, it did not show a statistically significant effect compared to Aandtaiji.

SUCRA ranking for the top three is in the order Aandtaiji (0.927), Qigong (0.671), and Taiji (0.615) (Table 2).

**Table 2:** Ranking table of interventions.

SUCRA					
Group	Intervention	MOCA	NO	MMSE	NO
MOCA.MMSE	Etaijigame	0.541	/	/	/
	Qigong	0.671	2	0.3139417	/
	QigongandT	0.438	/	0.40127	/
	Taiji	0.615	3	0.375725	/
	TaijitDCS	0.197	/	/	/
	Naoandtaiji	/	/	0.703	2
	Taijiandqigong	/	/	0.670	3
	Taijiball	/	/	0.992	1
	Aandtaiji	0.927	1	/	/
MOCA Subgroup MMSE Subgroup	BDJqigong	0.467	/	/	/
	BDJqigongandT	0.375	/	0.428	/
	YWLBqigong	0.449	/	0.324	/
	Eytaijigame	0.582	3	/	/
	LZJqigong	0.563	/	0.391	/
	SEDJqigong	0.871	1	/	/
	YFFqigong	0.494	/	/	/
	Ytaiji	0.560	/	0.392	/
	YtaijitDs	0.192	/	/	/
	Naoandytaiji	/	/	0.708	2
	Taijiandqigong	/	/	0.676	3
	Taijiball	/	/	0.983	1
	Aandytaiji	0.850	2	/	/

1. Qigong: Qi gong; 2. QigongandT: Qigong and Transcranial Direct Current Stimulation (tDCS) therapy; 3. Etaijigame: Exergaming-Based Tai Chi, a body movement-controlled computer Tai Chi game; 4. Taiji: Tai Chi or Tai ji; 5. Aandtaiji: Acupuncture and Tai ji; 6. TaijitDcs: Tai Chi combined with Transcranial Direct Current Stimulation (tDCS); 7. BDJqigong: Baduanjin Qigong; 8. BDJqigongandT: Baduanjin Qigong and Transcranial Direct Current Stimulation (tDCS) therapy; 9. LZJqigong: Liuzijue Qigong; 10. SEDJqigong: 12 Duanjin Qigong; 11. YFFqigong: Yifei Fang Qigong; 12. YWLBqigong: Yijinjing Qigong, Wuqinxi Qigong, Liuzijue Qigong, and Baduanjin Qigong; 13. EYtaijigame: Body movement-controlled computer Yang style Tai ji game; 14. Ytaiji: Yang style of Tai ji; 15. AandYtaiji: Acupuncture combined with Yang style Tai ji; 16. YtaijitDcs: Yang style Tai ji combined with Transcranial Direct Current Stimulation (tDCS); 17. Naoandtaiji: Nao Ling Tang and Tai ji; 18. Taijiandqigong: Tai ji and Qigong, organized by combining Yang-style and Wu-style Tai ji with Ba Duan Jin Qigong, Wu Qin Xi Qigong, and Liu Zi Jue Qigong; 19. Taijiball: Tai ji soft ball exercise; 20. NaoandYtaiji: Nao Ling Tang and Yang style of Tai ji. 21.SUCRA: Surface Under the Cumulative Ranking Curve.

### MOCA subgroup

Linkages between interventions: This section provides an explanation of the relationships between different interventions in the network meta-analysis. To compare which exercises had the greatest impact on cognitive function, traditional Chinese exercises (such as Tai Chi) were classified into MOCA subgroups based on their names (Supplementary Figure 2).

**Figure 4:**
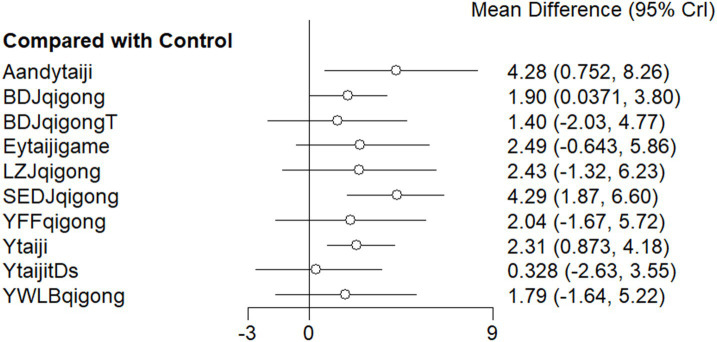
Comparison of MOCA subgroups intervention and control groups (Forest Plot). 1: Baduanjin Qigong + tDCS (BDJqigongandT); 2: Baduanjin Qigong (BDJqigong); 3: Liuzijue Qigong (LZJqigong); 4: Twelve Duanjin Qigong (SEDJqigong); 5: Yifei Fang Qigong (YFFqigong); 6: Yijinjing Qigong + Wuqinxi Qigong + Liuzijue Qigong + Baduanjin Qigong (YWLBqigong); 7: Yang-style Tai Ji game (EYtaijigame); 8: Yang-style Tai Ji (Ytaiji); 9: Acupuncture and Yang-style Tai Ji (AandYtaiji); 10: Yang-style Tai Ji combined with tDCS (YtaijitDcs). Method: The forest plot displays the mean differences between subgroup interventions and the control group, with 95% CrI. Results: Acupuncture with Yang-style Tai Ji (AandYtaiji) and Twelve Duanjin Qigong (SEDJqigong) showed significant improvements, while other interventions, such as Baduanjin Qigong, had smaller, non-significant effects.

For instance, BDJqigong and Ytaiji (Yang style Tai Chi) were directly compared with control groups in the previous studies. Other interventions combined Tai Chi or Qigong with additional treatments, such as YFFqigong and SEDJqigong, which had not been included in prior meta-analyses of traditional Chinese exercises and cognitive dysfunction. The figure illustrates a closed loop, indicating interconnected comparisons among interventions (Figure 4).

### Synthesized results

The results of MOCA subgroup network meta-analysis showed that compared with the control group, four interventions—SEDJqigong [RR = 4.29, 95% CI (1.87, 6.60), *p* < 0.05], Acupuncture combined with Yang style of Tai Chi [RR = 4.28, 95% CI (0.75, 8.26), *p* < 0.05], YTaiji [RR = 2.31, 95% CI (0.87, 4.18), *p* < 0.05], and BDJqigong (RR = 1.90, 95% CI (0.04, 3.80), *p* < 0.05)—bestowed significant advantages (Figure 4; Supplementary Table 3).

The top three SUCRA rankings were as follows: SEDJqigong (0.850), Aandyaiji (0.871), Eytaijigame (It is the body movement—controlled computer Yang style Tai Chi game) (0.581).

### The second index MMSE

Linkages between interventions: MMSE: 11 studies (Burgener et al., 2008; Rizhen et al., 2013; Tuan and Juan, 2013; Cheng et al., 2014; Li et al., 2014; Yong et al., 2016; Jun and Zhongxing, 2018; Siu and Lee, 2018; Lin et al., 2019; Jinping, 2022) took MMSE as the outcome index, among which the direct comparison between Taiji and the control group was the most, followed by the direct comparison between Qigong and the control group. Of these 11 studies, two included interventions (Rizhen et al., 2013; Jinping, 2022) that combined traditional Chinese exercises with other interventions. In addition, this study included Taijiball intervention measures that had never been included in traditional Chinese exercises and cognitive dysfunction in the previous meta-analysis. The figure does not show a closed loop (Supplementary Figure 3).

### Synthesized results

#### MMSE

**Figure 5:**
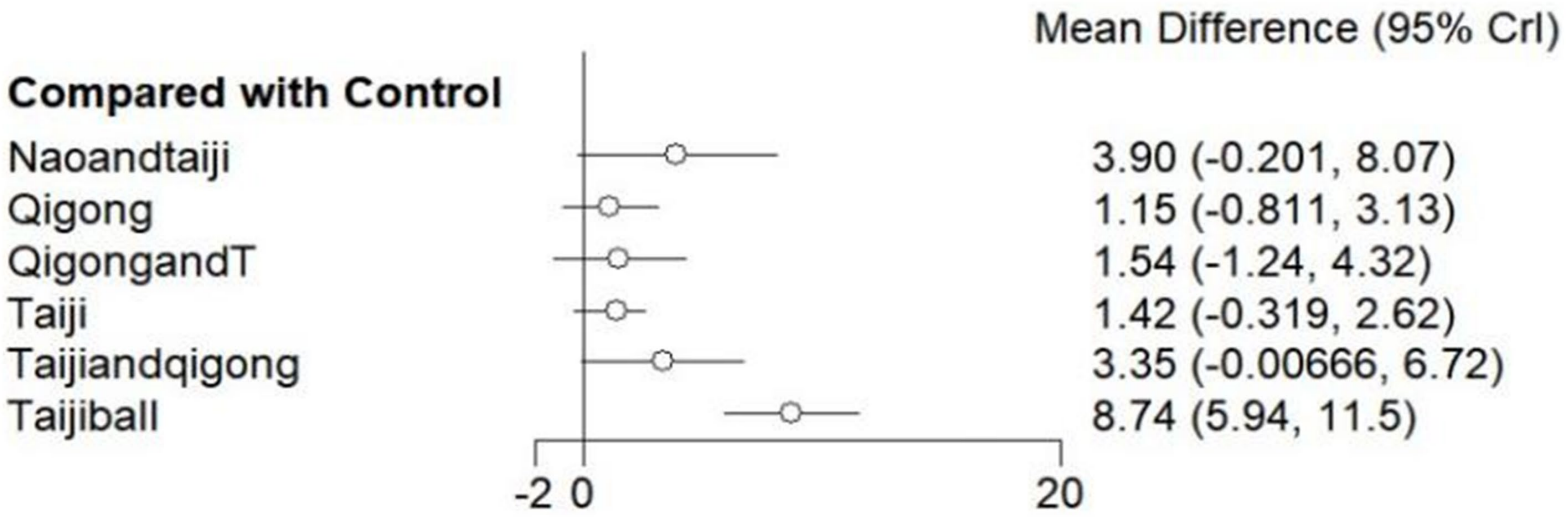
Comparison of MOCA subgroups intervention and control groups (Forest Plot). 1: Baduanjin Qigong + tDCS (BDJqigongandT); 2: Baduanjin Qigong (BDJqigong); 3: Liuzijue Qigong (LZJqigong); 4: Twelve Duanjin Qigong (SEDJqigong); 5: Yifei Fang Qigong (YFFqigong); 6: Yijinjing Qigong + Wuqinxi Qigong + Liuzijue Qigong + Baduanjin Qigong (YWLBqigong); 7: Yang-style Tai Ji game (EYtaijigame); 8: Yang-style Tai Ji (Ytaiji); 9: Acupuncture and Yang-style Tai Ji (AandYtaiji); 10: Yang-style Tai Ji combined with tDCS (YtaijitDcs). Method: The forest plot displays the mean differences between subgroup interventions and the control group, with 95% CrI. Results: Acupuncture with Yang-style Tai Ji (AandYtaiji) and Twelve Duanjin Qigong (SEDJqigong) showed significant improvements, while other interventions, such as Baduanjin Qigong, had smaller, non-significant effects.

The results of network meta-analysis showed that compared with the control group, Taijiball [RR = 8.74, 95% CI (5.94, 11.53), *p* < 0.05] had a significant advantage (Figure 5; Supplementary Table 4) The top three SUCRA rankings were as follows: Taijiball (0.992), Nao Ling Tang, and Tai Chi (Naoandtaiji) (0.703), and Taijiandqigong (Tai Chi Qigong is organized by combining Yang-style and Wu-style Tai Chi with Ba Duan Jin Qigong, Wu Qin Xi Qigong, and Liu Zi Jue Qigong) (0.670) (Table 2).

### MMSE subgroups

**Figure 6:**
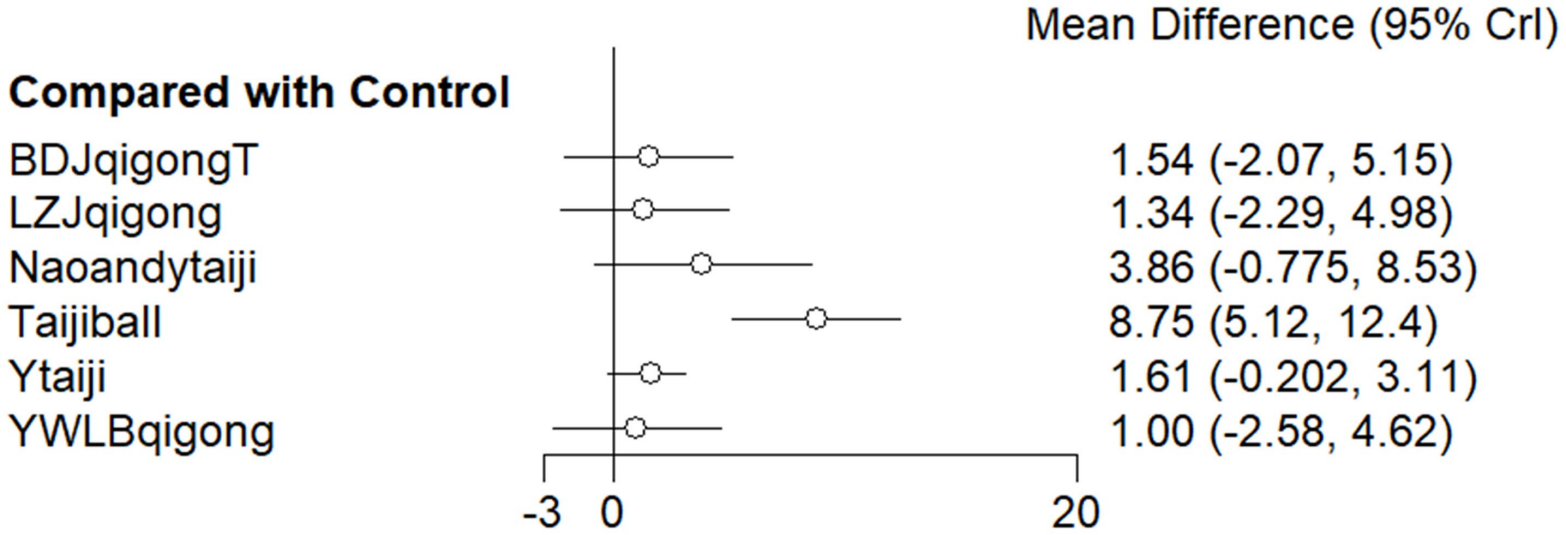
Comparison of MMSE subgroups intervention and control groups (Forest Plot). 1: Liuzijue Qigong (LZJqigong); 2: Nao Ling Tang and Yang-style Tai Ji (NaoanYtaiji); 3: Tai Ji and Qigong (combining Yang-style and Wu-style Tai Ji with Baduanjin, Wuqinxi, and Liuzijue Qigong); 4: Tai Ji soft ball exercise (Taijiball); 5: Yijinjing Qigong previous meta-analyses have limitations. Wuqinxi Qigong + Liuzijue Qigong + Baduanjin Qigong (YWLBqigong); 6: Yang-style Tai Ji (Ytaiji); 7: Baduanjin Qigong + tDCS (BDJQigongandT). Method: Mean difference analysis comparing various traditional exercises and a control group, with 95% CrI. Results: Tai Ji soft ball exercise (Taijiball) showed the greatest effect, while other interventions, such as Baduanjin Qigong with tDCS and Liuzijue Qigong, had smaller, non-significant effects.

The link between the interventions: There were studies that used a most direct comparison between Ytaiji and the control group, followed by the intervention combined with Qigong and other interventions combined with Ytaiji and the control group. The figure does not show a closed loop (Figure 6; Supplementary Table 5).

### Synthesized results

#### MMSE subgroups

Compared with the control group, there was still only one intervention, Taijiball [RR = 8.75, 95% CI (4.81, 12.68), *p* < 0.05] (Figure 6).

The top three in the SUCRA ranking are: Taijiball (0.983), Naoandytaiji (Nao Ling Tang and Yang style of Tai Chi) (0.708), Taijiandqigong (0.676) (Table 2).

Among them, the traditional Chinese exercises (such as Tai Chi and Qigong) ranked first: Taijiball (0.983). The intervention method combining traditional Chinese exercises (such as Tai Chi and qigong) with other means ranked first: Naoandytaiji (0.708). In MMSE subgroup, traditional Chinese exercises were superior to the intervention method of traditional Chinese exercises combined with other means.

### Meta-regression

Given the variability in the intervention durations (course) across different modalities, a meta-regression analysis was performed to assess the impact of intervention duration on MOCA and MMSE outcomes. This analysis aimed to quantify the extent to which intervention length influenced the respective cognitive performance measures. The results show that, compared with control group, Qigong [RR = 2.35, 95% CI (1.26, 3.36), *p* < 0.05], and Taiji [RR = 2.14, 95% CI (0.79, 3.72), *p* < 0.05], intervention time was correlated with MOCA index, the results were statistically significant, and the intervention time of other intervention measures had no significant correlation with MOCA index. From the MOCA subgroup analysis, the intervention time of Ytaiji [RR = 2.38, 95% CI (0.68, 4.48), *p* < 0.05] was related to the MOCA index, and the results were statistically significant. The intervention time of the rest of the interventions for the effects of MOCA indicators showed no significant correlation, and the results displayed no statistical significance. There was no significant correlation between the intervention time of different interventions and the MMSE indicators, and the results were not statistically significant (Table 2).

### Publication bias

**Figure 7:**
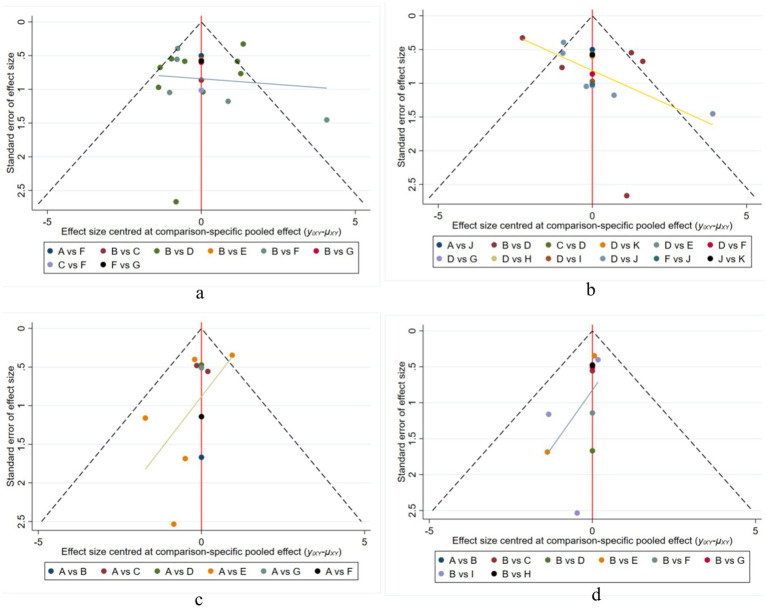
Funnel plots. **(a)** Funnel plot for MOCA indicators; **(b)** Funnel plot for MOCA subgroup indicators; **(c)** Funnel plot for MMSE indicators; **(d)** Funnel plot for MMSE subgroups. Method: Funnel plots display the effect sizes and standard errors of the included studies. Results: Some asymmetry was observed, indicating potential publication bias in certain comparisons.

Publication bias was assessed for the two indexes. As shown in the figure, there is little possibility of bias in the data (Figure 7).

## Discussion

### Summary of the main findings

A meta-analysis of 24 studies involving 1808 patients, including 13 RCTs on traditional Chinese exercises alone or combined with other treatments, showed Aandytaiji ranked first for MOCA scores overall, followed closely by SEDJqigong, though the latter had a small sample size and potential risk of bias. Taijiball showed the most significant improvement in MMSE scores. The meta-regression results indicated longer durations of Qigong and Tai Chi interventions yielded better outcomes (Supplementary Table 6).

### Interpretation of the results

First, this study confirms the efficacy of traditional Chinese exercises, alone or combined with other treatments, in treating cognitive impairment. The meta-analysis results are mixed. Lyu et al. (2021) found Tai Chi had no significant effect on cognitive dysfunction, while Zhou et al. (2022) reported significant improvements.

Secondly, several traditional Chinese exercises commonly used in research were not included in prior meta-analyses (Su et al., 2022; Zhou et al., 2022). This study is the first to examine the impact of traditional Chinese exercises, such as Tai Chi, on cognitive dysfunction within the context of a current meta-analysis. SEDJqigong (Jing et al., 2019; Hengjia et al., 2020), YFF qigong (Min and Xiaodan, 2023), and Taijiball (Yong et al., 2016) were among the interventions studied. Although SEDJqigong achieved the highest score in the MOCA subgroup analysis, its small sample size and potential risk of bias indicate that Aandytaiji remains the top-ranked intervention for MOCA overall. SEDJqigong, also known as Wenba Duan Jin (sitting Ba Duan Jin) (Lijiang, 2018), is a systemic exercise beneficial for both able-bodied individuals and those with lower limb disabilities (Lijiang, 2018; Tao, 2018). It involves cognitive functions such as memory, visuospatial ability, and executive ability and has a broader effect on cognitive function (Liu J. et al., 2024). In comparison, Aandytaiji enhances cognitive function and blood circulation, particularly in areas critical for cognition. Its combination of acupuncture and Tai Chi addresses ischemia, making it highly effective for early-stage dementia, further reinforcing its top ranking in the MOCA analysis (Romero-García et al., 2024; Subbarao et al., 2024). Taijiball ranked first in improving MMSE scores, and the limitations of previous meta-analyses were addressed in this analysis. In previous meta-analyses, Taijiball was not included as an intervention, limiting the assessment of traditional Chinese exercises on cognitive function, particularly MMSE scores. This study addresses that limitation by incorporating Taijiball, offering a more comprehensive perspective and explaining its strong performance in MMSE scores, providing new evidence of its contribution to cognitive function. Taijiball integrates soft, steady movements through the coordination of racket, ball, and body, providing a holistic workout (Wang et al., 2021). Continuous stimulation of acupuncture points on the hands during Taijiball practice activates the cerebral cortex, mitigating memory loss in the elderly. This supports Taijiball’s brain health benefits from a TCM perspective (Sun et al., 2021).

Additionally, previous randomized controlled trials typically compared traditional Chinese exercises with comprehensive interventions. A few studies included these combined interventions in meta-analyses. This study found that combined interventions improved MOCA scores more effectively than traditional exercises alone, while traditional exercises alone were more effective in improving MMSE scores. For MOCA scores, the combined intervention of acupuncture and Tai Chi ranked first. Pang et al. (2022) found that the frontal and temporal lobes are crucial for cognitive function. Acupuncture at Baihui and Zhisan (Shentingxue and Benshen), combined with Tai Chi, improved blood supply to these lobes, addressing ischemia and hypoperfusion (Yang et al., 2021). Tai Chi, based on Yin-Yang theory, may delay cognitive decline in elderly individuals with MCI and improve cognitive function in early-stage dementia (Wei et al., 2022).

For MOCA indicators, Aandytaiji ranks the highest. For MMSE indicators, Taijiball is highly effective, though its limited inclusion in the literature may introduce bias. Further research is needed to assess its full impact.

Although Tai Chi was included in most studies, its SUCRA ranking was not the highest. Various forms of Qigong were employed in prior studies, but RCTs focused mainly on Yang’s Tai Chi. There are many types of Tai Chi, each with unique characteristics. The limited number of studies on Chen’s Tai Chi and other forms may explain the suboptimal outcomes. More research is needed to address this gap (Joshi et al., 2024).

These findings support the positive role of traditional Chinese exercises, alone or combined with other treatments, in treating cognitive impairment. Comparing specific exercises can positively impact clinical treatment strategies and reduce medication costs for cognitive impairment patients.

## Discussion on heterogeneity

Variations in exercise protocols: This study examined different traditional Chinese exercises, such as Tai Chi, Qigong, and Taijiball, which differ significantly in practice. Tai Chi focuses on balance and gentle movements, while Qigong emphasizes breath regulation. These differences in duration, intensity, and frequency can lead to varying cognitive effects, increasing heterogeneity across studies (Cheng et al., 2024; Liu P. et al., 2024).

Participant characteristics: Participants vary by age, gender, and baseline cognitive impairment. Younger or healthier individuals may benefit more from interventions, while older adults or those with comorbidities like hypertension or diabetes may show weaker responses, contributing to variability in outcomes (Alford et al., 2024; Ibañez et al., 2024; Pramanik et al., 2024; Yin et al., 2024; Zhang L. et al., 2024).

Differences in study settings: Geographic and cultural factors also impact results. For example, participants in China may have higher adherence to Tai Chi due to cultural familiarity, while those in Western countries may be less engaged. Disparities in healthcare resources further affect intervention outcomes (Jimenez et al., 2024).

Study design and methodology: Variations in study design, including intervention duration, follow-up periods, and assessment methods, contribute to heterogeneity. Studies with shorter follow-ups may miss long-term effects, while longer studies may capture more significant cognitive changes (Wei et al., 2024; Ziccardi et al., 2024). Additionally, reliance on self-reported assessments versus clinical evaluations adds to variability.

### Limitations

This study included 24 articles, but several limitations could affect the reliability of the results. First, allocation concealment is critical for preventing selection bias, and a lack of transparency in this regard may reduce internal validity (Sharpe et al., 2024). Additionally, blinding was uncertain in some studies, introducing potential performance and detection biases, which could lead to an overestimation of the treatment effects (Choy et al., 2024). Second, small sample sizes in two studies reduced statistical power, increasing the risk of random error and possibly exaggerating treatment effects. Publication bias is also a concern, as positive results are more likely to be published, while negative or null findings may be underreported (Zhang et al., 2013). These limitations, particularly small sample sizes, lack of blinding, and potential publication bias, could result in overestimated effects and increased heterogeneity. Future research should focus on larger sample sizes, stricter blinding, and minimizing bias to improve the reliability and generalizability of the findings (Lin, 2018; Kulkarni et al., 2024; Tamrakar et al., 2024).

### Clinical implications and future directions

The findings of this study align with previous research while offering new insights. Aandtaiji and Taijiball showed strong performance in both MOCA and MMSE scores, suggesting their potential to improve cognitive function by regulating central nervous system activity (You and Ogawa, 2020). These results support traditional Chinese exercises, alone or combined with medical interventions, as effective strategies for enhancing brain function through energy balance, increased oxygenation, and neural activation (Gharpure et al., 2024; He et al., 2024). These findings have important implications for clinical practice. Incorporating traditional Chinese exercises into treatment plans can reduce medication dependency and improve outcomes for patients with cognitive impairment. As a low-cost, easily implemented intervention, traditional Chinese exercises are particularly suitable for managing long-term cognitive decline. They offer great potential in resource-limited settings, helping to ease the financial burden on healthcare systems and families while providing sustained cognitive benefits (Flores-Sandoval et al., 2024; Laakso et al., 2024).

## Conclusion

Research shows that traditional Chinese exercises, whether used alone or in combination with other treatments, can enhance cognitive abilities in patients with cognitive impairment. Combined treatments outperform traditional exercises alone in improving MOCA scores, whereas traditional exercises alone are more effective in enhancing MMSE scores. Additionally, longer practice durations of Tai Chi and Qigong lead to better results. Currently, despite the extensive research on Tai Chi as an intervention for cognitive impairment, the studies have primarily focused on a single form of Tai Chi, necessitating further in-depth research. This study is the first to include SEDJqigong and Taijiball, and more clinical trials are anticipated. These findings provide new evidence for the clinical treatment of cognitive impairment.

## Data Availability

The original contributions presented in the study are included in the article/Supplementary material, further inquiries can be directed to the corresponding author.

## Footnotes

^1^ https://www.R-project.org

## Supplementary material

Supplementary Figure 1 MOCA index intervention and control group network relationship graph. 1: Qigong; 2: Qigong and tDCS; 3: Exergaming-based Tai Ji (Etaijigame); 4: Tai Ji; 5: Acupuncture and Tai Ji (Aandtaiji); 6: Tai Ji combined with tDCS (TaijitDcs). Method: This network plot visualizes comparisons between interventions and the control group. Node size represents the sample size, and edge thickness reflects the number of studies. Results: The control group and Tai Ji had the most data, represented by larger nodes and thicker lines.

Supplementary Figure 2 MOCA subgroup intervention and control group network relationship graph. 1: Baduanjin Qigong + tDCS (BDJqigongandT); 2: Baduanjin Qigong (BDJqigong); 3: Liuzijue Qigong (LZJqigong); 4: Twelve Duanjin Qigong (SEDJqigong); 5: Yifei Fang Qigong (YFFqigong); 6: Yijinjing Qigong + Wuqinxi Qigong + Liuzijue Qigong + Baduanjin Qigong (YWLBqigong); 7: Yang-style Tai Ji game (EYtaijigame); 8: Yang-style Tai Ji (Ytaiji); 9: Acupuncture combined with Yang-style Tai Ji (AandYtaiji); 10: Yang-style Tai Ji combined with tDCS (YtaijitDcs). Method: A network plot displaying comparisons between subgroup interventions and the control group, with node size representing the sample size and edge thickness reflecting the number of comparisons. Results: Tai Ji and Qigong subgroups had the most comparisons with the control group.

Supplementary Figure 3 MMSE index intervention and control group network relationship graph. 1: Nao Ling Tang and Tai Ji (Naoantaiji); 2: Qigong and tDCS (QigongandT); 3: Exergaming-based Tai Ji (Etaijigame); 4: Tai Ji and Qigong (combining Yang-style and Wu-style Tai Ji with Baduanjin, Wuqinxi, and Liuzijue Qigong); 5: Tai Ji soft ball exercise (Taijiball). Method: Nodes represent interventions, and edges show direct comparisons between interventions and the control group. Results: The control group was most frequently compared with interventions such as Tai Ji and Qigong, indicated by larger nodes and thicker lines.

Supplementary Figure 4 MMSE subgroup intervention and control group network relationship graph. 1: Liuzijue Qigong (LZJqigong); 2: Nao Ling Tang and Yang-style Tai Ji (NaoanYtaiji); 3: Tai Ji and Qigong (combining Yang-style and Wu-style Tai Ji with Baduanjin, Wuqinxi, and Liuzijue Qigong); 4: Tai Ji soft ball exercise (Taijiball); 5: Yijinjing Qigong + Wuqinxi Qigong + Liuzijue Qigong + Baduanjin Qigong (YWLBqigong); 6: Yang-style Tai Ji (Ytaiji); 7: Baduanjin Qigong + tDCS (BDJQigongandT). Method: A network plot showing the relationship between various interventions and the control group. Results: The most frequent comparisons involved the control group and interventions such as Tai Ji and Qigong.
