# Supplementary material for: Effects of traditional Chinese exercises or their integration with medical treatments on cognitive impairment: a network meta-analysis based on randomized controlled trials
Source: Front Aging Neurosci. 2024 Nov 11;16:1475406. doi: 10.3389/fnagi.2024.1475406 (PMC11586772; doi:10.3389/fnagi.2024.1475406)
Supplement: Supplementary file 1 [file Table_1.docx]

Table 1. Characteristics of included studies in this review.

| **No** | **Title** | **language** | **Author** | **Country** | **Time** | **Patient** | **Intervention** | **Number** | **Course** | **Outcome** |
| --- | --- | --- | --- | --- | --- | --- | --- | --- | --- | --- |
| 1 | Tai Ji Quan and global cognitive function in older adults with cognitive impairment: A pilot study | English | Fuzhong Li | USA and CHINA | 2014 | MCI | 14-week Yang style tai Ji: Moving for Better Balance (TJQMBB) program ; | Taiji group( n=20 ) Control group (n = 20) | 14w | MMSE |
| 2 | Tai Chi Versus Conventional Exercise to Improve Cognitive Performance in Older Adults With Mild Cognitive Impairment | English | [Angus P Yu](https://pubmed.ncbi.nlm.nih.gov/?term=Yu+AP&cauthor_id=35614144) | HONG KONG, CHINA | 2022 | MCI | Tai Chi group: 24 weeks of Yang-style Tai Chi training three times a week for 60 minutes each session; Conventional Exercise group: 24 weeks of fitness training three times a week for 60 minutes each session; Control group: no intervention. | Tai Chi group(n=10) ； Exercise group(n=12)； Control group(n=.12) | 24w | MOCA |
| 3 | Tai chi qigong as a means to improve night-time sleep quality among older adults with cognitive impairment: A pilot randomized controlled trial | English | Aileen WK Chan | HONG KONG, CHINA | 2016 | MCI | Yang and Wu style tai Ji and Baduanjin,Wuqinxi,Liuzijue qigong sessions twice a week for 2 months, 60 minutes each session | Taiji group (n = 27 ）  Control group (n = 25) | 8w | MMSE |
| 4 | Simplified Tai Chi 6-Form Apparatus for Balance in Elderly People with Alzheimer's Disease | English | Lin, Y. C. | TAI WAN, CHINA | 2020 | MCI | The Yang style tai Ji group (TCGr) completed an 8-week training course for the Simplified Yang style tai Ji 6-Form Apparatus | Taiji group (n = 11 ） Control group (n = 10) | 8w | MMSE |
| 5 | Mind-Body Exercise Modulates Locus Coeruleus and Ventral Tegmental Area Functional Connectivity in Individuals With Mild Cognitive Impairment | English | Liu Jiao | CHINA | 2021 | MCI | Participants were randomized into Qigong, brisk walking, or a healthy education control group for 6 months. The exercise groups participated in sessions 3 days/week, 60 min/day. | Qigong group (n = 20) Control group (n = 20) | 24w | MOCA |
| 6 | Mental and Physical Activities Delay  Cognitive Decline in Older Persons  with Dementi | English | Jing Tao ; | Hong Kong CHINA | 2014 | Early to early-middle stages of dementia | mahjong. Taiji group. Control group | mahjong（n=36） Taiji group (n = 39) Control group (n = 35) | 24w | MOCA |
| 7 | Effects of Tai Chi on cognition and instrumental activities of daily living in community dwelling older people with mild cognitive impairment | English | Siu, Mei Yi | Hong Kong CHINA | 2017 | MCI | The intervention group received 16 weeks of Yang-style Tai Chi training, two sessions per week, each lasting one hour | Taiji group (n = 80)  Control group (n= 80) | 16w | MMSE |
| 8 | Effects of Tai Chi combined with tDCS on cognitive function in patients with MCI: a randomized controlled trial | English | Ying Xu | USA and CHINA | 2023 | MCI | Taiji and tDCS.Tai Ji. ontrol | Taiji and tDCS(n=44 ) Tai Ji(n=49) ontrol group (n = 44) | 24w | MOCA |
| 9 | Effects of Tai Chi Chuan on Cognitive Function in Adults 60 Years or Older With Type 2 Diabetes and Mild Cognitive Impairment in China: A Randomized Clinical Trial | English | Yannan Chen | USA and CHINA | 2023 | MCI | Yang style tai Ji and walking training, both for 60 min/session, 3 times/wk for 24 weeks | Taiji group (n = 110) Control group (n = 110) | 36w | MOCA |
| 10 | Effects of mind-body exercise baduanjin on cognition in community-dwelling older people with mild cognitive impairment: a randomized controlled trial | English | Xia Rui | CHINA | 2022 | MCI | Baduanjin exercise group received 24 weeks of Baduanjin exercise training, 60 min sessions, 3 days per week; Brisk walking group received 24 weeks of brisk walking, 60 min per session, 3 sessions per week / | Qigong group (n = 70) Control group (n = 65) | 24w | MOCA |
| 11 | Effects of exergaming based Tai Chi on Cognition and dual task gait in older adults with mild cognitive Impairment a randomized control trial | English | Chen Liang Liu | CHINA | 2022 | MCI | EXER-TC and TC groups received 36 training sessions (three 50-min sessions per week) for 12 weeks | EXER-taiji(n=16Taiji group(n=17) Control group (n = 19) | 12W | MOCA |
| 12 | The effects of a multimodal intervention on outcomes of persons with early-stage dementia | English | Sandy C. Burgener | USA | 2008 | Early to early-middle stages of dementia | 40-week intervention, including Taiji exercises, and support group participation | Taiji group (n = 24)  Control group (n = 19) | 40w | MMSE |
| 13 | Clinical study of acupuncture combined with Tai Chi in the treatment of mild cognitive impairment caused by cerebral small vessel disease | Chinese | Ze Yu Shen | CHINA | 2021 | MCI | Acupuncture combined with Yang style tai Ji intervention; the control group used only Yang style tai Ji intervention. | A+Taiji(n = 31)；  Control group (n = 31) | 8w. | MOCA |
| 14 | A study on the evaluation of the clinical efficacy of Baduanjin in patients with mild cognitive dysfunction | Chinese | Qian Yang | CHINA | 2019 | MCI | Ba duan jin qigong 5 times a week, 40 minutes each session, for 24 weeks. The control group received no intervention | Qigong group (n = 32) Control group (n = 32) | 24w | MOCA |
| 15 | The effect of Naoling decoction combined with Tai Chi on the rehabilitation of patients with Alzheimer's disease | Chinese | Ri Zhen Li | CHINA | 2013 | Early-stage Alzheimer's disease | he treatment group received Naoling Decoction combined with Yang style tai Ji exercise, while the control group received conventional treatment | Naoling +Ytaiji(n = 32) Control group (n = 30) | 12w | MMSE |
| 16 | Study on the Rehabilitation Effects of Six-Character Formula of Health Qigong on Mild Cognitive Impairment in the Elderly | Chinese | Xin Tuan Zheng | CHINA | 2013 | MCI | "Six Healing Sounds" fitness Qigong exercise, practiced twice daily for 30 minutes each session, 5 days a week for 6 months | Qigong group (n = 45) Control group (n = 43) | 24w | MOCA |
| 17 | The Impact of Virtual Reality-Based Baduanjin Exercise on Mild Cognitive Impairment in Elderly Patients in Nursing Homes | Chinese | Sun Zhi Chen | CHINA | 2021 | MCI | VR-based qigong 50 minutes per session, three times a week for 24 weeks | VR qigong (n = 29) Control group (n = 28) | 24w | MOCA |
| 18 | Study on the Intervention Effect of Continuous Health Qigong Exercise on Mild Cognitive Impairment in the Elderly | Chinese | Jun Cai | CHINA | 2018 | MCI | 6 months of fitness Qigong exercises including Yi Jin Jing, Ba Duan Jin, Wu Qin Xi, Liu Zi Jue | Qigong group (n = 28), Control group (n = 30) | 24w | MOCA,MMSE |
| 19 | Clinical Observation of the Efficacy of Eight-Style Tai Chi on Mild Cognitive Impairment Caused by Lacunar Infarction | Chinese | Mei Ling Huang | CHINA | 2021 | MCI | Yang style tai Ji group practiced Eight Style Taijiquan for 30 minutes | Taiji group (n = 33) Control group (n = 33) | 24w | MOCA |
| 20 | Clinical Study of Baduanjin Combined with Transcranial Direct Current Stimulation in Treating Mild Cognitive Impairment After Stroke | Chinese | Jin Ping Feng | CHINA | 2022 | MCI | The control group received transcranial direct current stimulation, while the observation group Baduanjin exercise | T+qigong (n = 47) Control group (n = 47) | 8w | MOCA,MMSE |
| 21 | The Impact of Baduanjin on the Cognitive Levels of Patients with Mild Cognitive Impairment | Chinese | Tao Liu | CHINA | 2018 | MCI | Baduanjin exercise intervention for 6 months | Qigong group (n = 30） Control group (n = 30) | 24w | MOCA |
| 22 | Experimental Study on Tai ji Soft Ball Exercise in the Treatment of Senile Dementia | Chinese | Yong Zhou | CHINA | 2016 | Early to early-middle stages of dementia | Taijiball(softball) exercise under professional guidance, compared to jogging in the control group | Taijiball group (n =18) Control group (n = 18) | 32w | MMSE |
| 23 | Study on the Impact and Mechanism of Health Qigong Yangfei Prescription on Cognitive Function in Patients with Stable Chronic Obstructive Pulmonary Disease | Chinese | Min Zhuang | CHINA | 2023 | Severity levels GOLD 1-3 (FEV1≥30%) | Yangfeifang Qigong exercise, 5 days a week, 2 sessions per day, 35 minutes per session for 12 weeks | Qigong group (n = 18)  Control group (n = 18) | 12w | MOCA |
| 24 | 59.Study on the Impact of Health Qigong Twelve Duanjin on Patients with Mild Cognitive Impairment | Chinese | Heng Jia Liu | CHINA | 2020 | MCI | Twelve Dan Jin qigong Exercises performed 5 times per week, 40 minutes per session, over 24 weeks | Qigong group (n = 30) Control group (n = 30) | 24w | MOCA |

Table 2. Ranking table of interventions

| SUCRA | | | | | |
| --- | --- | --- | --- | --- | --- |
| **Group** | **Intervention** | **MOCA** | **NO** | **MMSE** | **NO** |
| MOCA.MMSE | Etaijigame | 0.541 | / | / | / |
|  | Qigong | 0.671 | 2 | 0.3139417 | / |
|  | QigongandT | 0.438 | / | 0.40127 | / |
|  | Taiji | 0.615 | **3** | 0.375725 | / |
|  | TaijitDCS | 0.197 | / | / | / |
|  | Naoandtaiji | / | / | 0.703 | 2 |
|  | Taijiandqigong | / | / | 0.670 | 3 |
|  | Taijiball | / | / | 0.992 | 1 |
|  | Aandtaiji | 0.927 | 1 | / | / |
| MOCA Subgroup  MMSE Subgroup | BDJqigong | 0.467 | / | / | / |
|  | BDJqigongandT | 0.375 | / | 0.428 | / |
|  | YWLBqigong | 0.449 | / | 0.324 | / |
|  | Eytaijigame | 0.582 | 3 | / | / |
|  | LZJqigong | 0.563 | / | 0.391 | / |
|  | SEDJqigong | 0.871 | 1 | / | / |
|  | YFFqigong | 0.494 | / | / | / |
|  | Ytaiji | 0.560 | / | 0.392 | / |
|  | YtaijitDs | 0.192 | / | / | / |
|  | Naoandytaiji | / | / | 0.708 | 2 |
|  | Taijiandqigong | / | / | 0.676 | 3 |
|  | Taijiball | / | / | 0.983 | 1 |
|  | Aandytaiji | 0.850 | 2 | / | / |

1.Qigong: Qi gong; 2. QigongandT: Qigong and Transcranial Direct Current Stimulation (tDCS) therapy; 3. Etaijigame: Exergaming-Based Tai Chi, a body movement-controlled computer Tai Chi game; 4. Taiji: Tai Chi or Tai ji; 5. Aandtaiji: Acupuncture and Tai ji; 6. TaijitDcs: Tai Chi combined with Transcranial Direct Current Stimulation (tDCS); 7. BDJqigong: Baduanjin Qigong; 8. BDJqigongandT: Baduanjin Qigong and Transcranial Direct Current Stimulation (tDCS) therapy; 9. LZJqigong: Liuzijue Qigong; 10. SEDJqigong: Twelve Duanjin Qigong; 11. YFFqigong: Yifei Fang Qigong; 12. YWLBqigong: Yijinjing Qigong, Wuqinxi Qigong, Liuzijue Qigong, and Baduanjin Qigong; 13. EYtaijigame: Body movement-controlled computer Yang style Tai ji game; 14. Ytaiji: Yang style of Tai ji; 15. AandYtaiji: Acupuncture combined with Yang style Tai ji; 16. YtaijitDcs: Yang style Tai ji combined with Transcranial Direct Current Stimulation (tDCS); 17. Naoandtaiji: Nao Ling Tang and Tai ji; 18. Taijiandqigong: Tai ji and Qigong, organized by combining Yang-style and Wu-style Tai ji with Ba Duan Jin Qigong, Wu Qin Xi Qigong, and Liu Zi Jue Qigong; 19. Taijiball: Tai ji soft ball exercise; 20. NaoandYtaiji: Nao Ling Tang and Yang style of Tai ji. 21.SUCRA:Surface Under the Cumulative Ranking Curve

Supplement Table 1 literature search

|  | Query | Search Details | Results | Time |
| --- | --- | --- | --- | --- |
| 4 | (((((((((((((Tai Ji[MeSH Terms]) OR (Tai Chi[Title/Abstract])） OR (Tai Chi soft ball exercise[Title/Abstract])) OR (Chen style Tai Chi [Title/Abstract])) OR (Yang style Tai Chi [Title/Abstract])) OR (Sun style Tai Chi [Title/Abstract])) OR (Wu style Tai Chi [Title/Abstract])) OR (Tai-ji[Title/Abstract])) OR (Chi, Tai[Title/Abstract])) OR (Tai Ji Quan[Title/Abstract])) OR (Ji Quan, Tai[Title/Abstract])) OR (Quan, Tai Ji[Title/Abstract])) OR (Taiji[Title/Abstract])) OR (T'ai Chi[Title/Abstract])) OR (Tai Chi Chuan[Title/Abstract])) OR (Taiji quan[Title/Abstract])) OR (Taijiquan[Title/Abstract])) OR ((((((((((((((((((((Qigong[MeSH Terms]) OR (Qi Gong[Title/Abstract])) OR(Yangfeifangqigong[Title/Abstract])) OR (Twelve Dan Jin qigong[Title/Abstract])) OR (Ch'i Kung[Title/Abstract])) OR (chi kung[Title/Abstract])) OR (chigung[Title/Abstract])) OR (qi gong[Title/Abstract])) OR (qigong exercise[Title/Abstract])) OR (Eight Pieces of Brocade[Title/Abstract])) OR (Baduanjin[Title/Abstract])) OR (Muscle Changing Classic[Title/Abstract])) OR (Yijinjing[Title/Abstract])) OR (Five Animal Frolics[Title/Abstract])) OR (Wuqinxi[Title/Abstract])) OR (Six-Character Formula[Title/Abstract])) OR (Liuzijue[Title/Abstract])) OR (Guiding[Title/Abstract] AND Pulling Techniques[Title/Abstract])) OR (Daoyinshu[Title/Abstract])) OR (Chinese Kung Fu[Title/Abstract]))) AND ((((((((((((((((((((((((((((((((((((Cognitive Dysfunction[MeSH Terms]) OR (cognitive defect[Title/Abstract])) OR (Cognitive Dysfunctions[Title/Abstract])) OR (Dysfunction, Cognitive[Title/Abstract])) OR (Dysfunctions, Cognitive[Title/Abstract])) OR (Cognitive Impairments[Title/Abstract])) OR (Impairment, Cognitive[Title/Abstract])) OR (Impairments, Cognitive[Title/Abstract])) OR (Disorder, Cognitive[Title/Abstract])) OR (Disorders, Cognitive[Title/Abstract])) OR (Mild Cognitive Impairment[Title/Abstract])) OR (Cognitive Impairment, Mild[Title/Abstract])) OR (Cognitive Impairments, Mild[Title/Abstract])) OR (?Impairment, Mild Cognitive[Title/Abstract])) OR (Impairments, Mild Cognitive[Title/Abstract])) OR (Mild Cognitive Impairments[Title/Abstract])) OR (Cognitive Decline[Title/Abstract])) OR (Cognitive Declines[Title/Abstract])) OR (Decline, Cognitive[Title/Abstract])) OR (Declines, Cognitive[Title/Abstract])) OR (Mental Deterioration[Title/Abstract])) OR (Deterioration, Mental[Title/Abstract])) OR (Deteriorations, Mental[Title/Abstract])) OR (Mental Deteriorations[Title/Abstract])) OR (cognition disorder[Title/Abstract])) OR (cognition disorders[Title/Abstract])) OR (cognitive defects[Title/Abstract])) OR (cognitive deficit[Title/Abstract])) OR (cognitive disability[Title/Abstract])) OR (cognitive disorder[Title/Abstract])) OR (cognitive disorders[Title/Abstract])) OR (cognitive impairment[Title/Abstract])) OR (delirium, dementia, amnestic, cognitive[Title/Abstract])) OR (disorders[Title/Abstract])) OR (overinclusion[Title/Abstract])) OR (response interference[Title/Abstract])) | ("Tai-ji"[MeSH Terms] OR "tai chi"[Title/Abstract] OR ((("Tai-ji"[MeSH Terms] OR ("Tai"[All Fields] AND "Ji"[All Fields]) OR "Tai-ji"[All Fields] OR ("Tai"[All Fields] AND "Chi"[All Fields]) OR "tai chi"[All Fields]) AND "soft"[All Fields]) AND "ball exercise"[Title/Abstract]) OR "chen style tai chi"[Title/Abstract] OR "yang style tai chi"[Title/Abstract] OR "sun style tai chi"[Title/Abstract] OR "wu style tai chi"[Title/Abstract] OR "Tai-ji"[Title/Abstract] OR "chi tai"[Title/Abstract] OR "tai ji quan"[Title/Abstract] OR "ji quan tai"[Title/Abstract] OR "quan tai ji"[Title/Abstract] OR "Taiji"[Title/Abstract] OR "t ai chi"[Title/Abstract] OR "tai chi chuan"[Title/Abstract] OR "taiji quan"[Title/Abstract] OR "Taijiquan"[Title/Abstract] OR ("qigong"[MeSH Terms] OR "qi gong"[Title/Abstract] OR (("Twelve"[All Fields] AND ((jin, dan[Author] OR dan, jin[Author]) OR dan jin[Author])) AND "qigong"[Title/Abstract]) OR "ch i kung"[Title/Abstract] OR "chi kung"[Title/Abstract] OR "chigung"[Title/Abstract] OR "qi gong"[Title/Abstract] OR "qigong exercise"[Title/Abstract] OR ((("eight"[All Fields] OR "eights"[All Fields]) AND ("piece"[All Fields] OR "pieced"[All Fields] OR "pieces"[All Fields] OR "piecing"[All Fields])) AND "Brocade"[Title/Abstract]) OR "Baduanjin"[Title/Abstract] OR ((("muscle s"[All Fields] OR "muscles"[MeSH Terms] OR "muscles"[All Fields] OR "muscle"[All Fields]) AND ("change"[All Fields] OR "changed"[All Fields] OR "changes"[All Fields] OR "changing"[All Fields] OR "changings"[All Fields])) AND "Classic"[Title/Abstract]) OR "Yijinjing"[Title/Abstract] OR "five animal frolics"[Title/Abstract] OR "Wuqinxi"[Title/Abstract] OR "six character formula"[Title/Abstract] OR "Liuzijue"[Title/Abstract] OR ("Guiding"[Title/Abstract] AND "pulling techniques"[Title/Abstract]) OR "Daoyinshu"[Title/Abstract] OR "chinese kung fu"[Title/Abstract])) AND ("cognitive dysfunction"[MeSH Terms] OR "cognitive defect"[Title/Abstract] OR "cognitive dysfunctions"[Title/Abstract] OR "dysfunction cognitive"[Title/Abstract] OR "dysfunctions cognitive"[Title/Abstract] OR "cognitive impairments"[Title/Abstract] OR "impairment cognitive"[Title/Abstract] OR "impairments cognitive"[Title/Abstract] OR "disorder cognitive"[Title/Abstract] OR "disorders cognitive"[Title/Abstract] OR "mild cognitive impairment"[Title/Abstract] OR "cognitive impairment mild"[Title/Abstract] OR "cognitive impairments mild"[Title/Abstract] OR "impairment mild cognitive"[Title/Abstract] OR "impairments mild cognitive"[Title/Abstract] OR "mild cognitive impairments"[Title/Abstract] OR "cognitive decline"[Title/Abstract] OR "cognitive declines"[Title/Abstract] OR "decline cognitive"[Title/Abstract] OR "declines cognitive"[Title/Abstract] OR "mental deterioration"[Title/Abstract] OR "deterioration mental"[Title/Abstract] OR (("deteriorate"[All Fields] OR "deteriorated"[All Fields] OR "deteriorates"[All Fields] OR "deteriorating"[All Fields] OR "Deterioration"[All Fields] OR "Deteriorations"[All Fields] OR "deteriorative"[All Fields]) AND "Mental"[Title/Abstract]) OR "mental deteriorations"[Title/Abstract] OR "cognition disorder"[Title/Abstract] OR "cognition disorders"[Title/Abstract] OR "cognitive defects"[Title/Abstract] OR "cognitive deficit"[Title/Abstract] OR "cognitive disability"[Title/Abstract] OR "cognitive disorder"[Title/Abstract] OR "cognitive disorders"[Title/Abstract] OR "cognitive impairment"[Title/Abstract] OR "delirium dementia amnestic cognitive"[Title/Abstract] OR "Disorders"[Title/Abstract] OR "overinclusion"[Title/Abstract] OR "response interference"[Title/Abstract]) | 388 | 8:09:11 |
| 3 | (((((((((((((((((((Qigong[MeSH Terms]) OR (Qi Gong[Title/Abstract])) OR(Yangfeifangqigong[Title/Abstract])) OR (Twelve Dan Jin qigong[Title/Abstract])) OR (Ch'i Kung[Title/Abstract])) OR (chi kung[Title/Abstract])) OR (chigung[Title/Abstract])) OR (qi gong[Title/Abstract])) OR (qigong exercise[Title/Abstract])) OR (Eight Pieces of Brocade[Title/Abstract])) OR (Baduanjin[Title/Abstract])) OR (Muscle Changing Classic[Title/Abstract])) OR (Yijinjing[Title/Abstract])) OR (Five Animal Frolics[Title/Abstract])) OR (Wuqinxi[Title/Abstract])) OR (Six-Character Formula[Title/Abstract])) OR (Liuzijue[Title/Abstract])) OR (Guiding[Title/Abstract] AND Pulling Techniques[Title/Abstract])) OR (Daoyinshu[Title/Abstract])) OR (Chinese Kung Fu[Title/Abstract]) | "qigong"[MeSH Terms] OR "qi gong"[Title/Abstract] OR (("Twelve"[All Fields] AND ((jin, dan[Author] OR dan, jin[Author]) OR dan jin[Author])) AND "qigong"[Title/Abstract]) OR "ch i kung"[Title/Abstract] OR "chi kung"[Title/Abstract] OR "chigung"[Title/Abstract] OR "qi gong"[Title/Abstract] OR "qigong exercise"[Title/Abstract] OR ((("eight"[All Fields] OR "eights"[All Fields]) AND ("piece"[All Fields] OR "pieced"[All Fields] OR "pieces"[All Fields] OR "piecing"[All Fields])) AND "Brocade"[Title/Abstract]) OR "Baduanjin"[Title/Abstract] OR ((("muscle s"[All Fields] OR "muscles"[MeSH Terms] OR "muscles"[All Fields] OR "muscle"[All Fields]) AND ("change"[All Fields] OR "changed"[All Fields] OR "changes"[All Fields] OR "changing"[All Fields] OR "changings"[All Fields])) AND "Classic"[Title/Abstract]) OR "Yijinjing"[Title/Abstract] OR "five animal frolics"[Title/Abstract] OR "Wuqinxi"[Title/Abstract] OR "six character formula"[Title/Abstract] OR "Liuzijue"[Title/Abstract] OR ("Guiding"[Title/Abstract] AND "pulling techniques"[Title/Abstract]) OR "Daoyinshu"[Title/Abstract] OR "chinese kung fu"[Title/Abstract] | 1,553 | 8:06:49 |
| 2 | (((((((((((Tai Ji[MeSH Terms]) OR (Tai Chi[Title/Abstract])） OR (Tai Chi soft ball exercise[Title/Abstract])) OR (Chen style Tai Chi [Title/Abstract])) OR (Yang style Tai Chi [Title/Abstract])) OR (Sun style Tai Chi [Title/Abstract])) OR (Wu style Tai Chi [Title/Abstract])) OR (Tai-ji[Title/Abstract])) OR (Chi, Tai[Title/Abstract])) OR (Tai Ji Quan[Title/Abstract])) OR (Ji Quan, Tai[Title/Abstract])) OR (Quan, Tai Ji[Title/Abstract])) OR (Taiji[Title/Abstract])) OR (T'ai Chi[Title/Abstract])) OR (Tai Chi Chuan[Title/Abstract])) OR (Taiji quan[Title/Abstract])) OR (Taijiquan[Title/Abstract]) | "Tai-ji"[MeSH Terms] OR "tai chi"[Title/Abstract] OR ((("Tai-ji"[MeSH Terms] OR ("Tai"[All Fields] AND "Ji"[All Fields]) OR "Tai-ji"[All Fields] OR ("Tai"[All Fields] AND "Chi"[All Fields]) OR "tai chi"[All Fields]) AND "soft"[All Fields]) AND "ball exercise"[Title/Abstract]) OR "chen style tai chi"[Title/Abstract] OR "yang style tai chi"[Title/Abstract] OR "sun style tai chi"[Title/Abstract] OR "wu style tai chi"[Title/Abstract] OR "Tai-ji"[Title/Abstract] OR "chi tai"[Title/Abstract] OR "tai ji quan"[Title/Abstract] OR "ji quan tai"[Title/Abstract] OR "quan tai ji"[Title/Abstract] OR "Taiji"[Title/Abstract] OR "t ai chi"[Title/Abstract] OR "tai chi chuan"[Title/Abstract] OR "taiji quan"[Title/Abstract] OR "Taijiquan"[Title/Abstract] | 2,878 | 8:04:46 |
| 1 | (((((((((((((((((((((((((((((((((((Cognitive Dysfunction[MeSH Terms]) OR (cognitive defect[Title/Abstract])) OR (Cognitive Dysfunctions[Title/Abstract])) OR (Dysfunction, Cognitive[Title/Abstract])) OR (Dysfunctions, Cognitive[Title/Abstract])) OR (Cognitive Impairments[Title/Abstract])) OR (Impairment, Cognitive[Title/Abstract])) OR (Impairments, Cognitive[Title/Abstract])) OR (Disorder, Cognitive[Title/Abstract])) OR (Disorders, Cognitive[Title/Abstract])) OR (Mild Cognitive Impairment[Title/Abstract])) OR (Cognitive Impairment, Mild[Title/Abstract])) OR (Cognitive Impairments, Mild[Title/Abstract])) OR (?Impairment, Mild Cognitive[Title/Abstract])) OR (Impairments, Mild Cognitive[Title/Abstract])) OR (Mild Cognitive Impairments[Title/Abstract])) OR (Cognitive Decline[Title/Abstract])) OR (Cognitive Declines[Title/Abstract])) OR (Decline, Cognitive[Title/Abstract])) OR (Declines, Cognitive[Title/Abstract])) OR (Mental Deterioration[Title/Abstract])) OR (Deterioration, Mental[Title/Abstract])) OR (Deteriorations, Mental[Title/Abstract])) OR (Mental Deteriorations[Title/Abstract])) OR (cognition disorder[Title/Abstract])) OR (cognition disorders[Title/Abstract])) OR (cognitive defects[Title/Abstract])) OR (cognitive deficit[Title/Abstract])) OR (cognitive disability[Title/Abstract])) OR (cognitive disorder[Title/Abstract])) OR (cognitive disorders[Title/Abstract])) OR (cognitive impairment[Title/Abstract])) OR (delirium, dementia, amnestic, cognitive[Title/Abstract])) OR (disorders[Title/Abstract])) OR (overinclusion[Title/Abstract])) OR (response interference[Title/Abstract]) | "cognitive dysfunction"[MeSH Terms] OR "cognitive defect"[Title/Abstract] OR "cognitive dysfunctions"[Title/Abstract] OR "dysfunction cognitive"[Title/Abstract] OR "dysfunctions cognitive"[Title/Abstract] OR "cognitive impairments"[Title/Abstract] OR "impairment cognitive"[Title/Abstract] OR "impairments cognitive"[Title/Abstract] OR "disorder cognitive"[Title/Abstract] OR "disorders cognitive"[Title/Abstract] OR "mild cognitive impairment"[Title/Abstract] OR "cognitive impairment mild"[Title/Abstract] OR "cognitive impairments mild"[Title/Abstract] OR "impairment mild cognitive"[Title/Abstract] OR "impairments mild cognitive"[Title/Abstract] OR "mild cognitive impairments"[Title/Abstract] OR "cognitive decline"[Title/Abstract] OR "cognitive declines"[Title/Abstract] OR "decline cognitive"[Title/Abstract] OR "declines cognitive"[Title/Abstract] OR "mental deterioration"[Title/Abstract] OR "deterioration mental"[Title/Abstract] OR (("deteriorate"[All Fields] OR "deteriorated"[All Fields] OR "deteriorates"[All Fields] OR "deteriorating"[All Fields] OR "Deterioration"[All Fields] OR "Deteriorations"[All Fields] OR "deteriorative"[All Fields]) AND "Mental"[Title/Abstract]) OR "mental deteriorations"[Title/Abstract] OR "cognition disorder"[Title/Abstract] OR "cognition disorders"[Title/Abstract] OR "cognitive defects"[Title/Abstract] OR "cognitive deficit"[Title/Abstract] OR "cognitive disability"[Title/Abstract] OR "cognitive disorder"[Title/Abstract] OR "cognitive disorders"[Title/Abstract] OR "cognitive impairment"[Title/Abstract] OR "delirium dementia amnestic cognitive"[Title/Abstract] OR "Disorders"[Title/Abstract] OR "overinclusion"[Title/Abstract] OR "response interference"[Title/Abstract] | 1,075,570 | 7:59:29 |

Supplement Table 2. MOCA index intervention and league table of control group

| **RR(95%CI)** | | | | | | | |
| --- | --- | --- | --- | --- | --- | --- | --- |
|  | Aandtaiji | Control | Etaijigame | Qigong | QigongandT | Taiji | TaijitDCS |
| Aandtaiji | Aandtaiji | -4.11 (-7.46, -1.02) | -2.25 (-6.37, 1.8) | -1.77 (-5.34, 1.47) | -2.73 (-7.2, 1.51) | -1.98 (-4.85, 0.92) | -3.88 (-7.87, -0.01) |
| Control | 4.11 (1.02, 7.46)* | Control | 1.87 (-0.92, 4.82) | 2.35 (1.26, 3.36) | 1.39 (-1.54, 4.33) | 2.14 (0.79, 3.72) | 0.24 (-2.36, 2.99) |
| Etaijigame | 2.25 (-1.8, 6.37) | -1.87 (-4.82, 0.92) | Etaijigame | 0.47 (-2.7, 3.42) | -0.48 (-4.64, 3.52) | 0.27 (-2.59, 3.21) | -1.62 (-5.45, 2.14) |
| Qigong | 1.77 (-1.47, 5.34) | -2.35 (-3.36, -1.26) | -0.47 (-3.42, 2.7) | Qigong | -0.96 (-4.02, 2.21) | -0.2 (-1.87, 1.77) | -2.11 (-4.88, 0.88) |
| QigongandT | 2.73 (-1.51, 7.2) | -1.39 (-4.33, 1.54) | 0.48 (-3.52, 4.64) | 0.96 (-2.21, 4.02) | QigongandT | 0.75 (-2.4, 4.13) | -1.14 (-5.07, 2.87) |
| Taiji | 1.98 (-0.92, 4.85) | -2.14 (-3.72, -0.79) | -0.27 (-3.21, 2.59) | 0.2 (-1.77, 1.87) | -0.75 (-4.13, 2.4) | Taiji | -1.9 (-4.63, 0.72) |
| TaijitDCS | 3.88 (0.01, 7.87)* | -0.24 (-2.99, 2.36) | 1.62 (-2.14, 5.45) | 2.11 (-0.88, 4.88) | 1.14 (-2.87, 5.07) | 1.9 (-0.72, 4.63) | TaijitDCS |

1: Qigong:Qi gong ; 2: QigongandT: Qigong and Transcranial direct current stimulation therapy ; 3: Etaijigame: Exergaming-Based Tai Chi is body movement-controlled computer Tai Chi game; 4: Taiji: Tai Chi or Tai ji; 5: Aandtaiji: Acupuncture and Tai ji; 6: TaijitDcs: Tai Chi combined with tDCS (transcranial Direct Current Stimulation); 7: RR: Risk Ratio; 8: CI: Confidence Interval.

*: The data is statistically significant

Supplement Table 3. MOCA subgroups index intervention and league table of control group

| **RR(95%CI)** | | | | | | | | | | | |
| --- | --- | --- | --- | --- | --- | --- | --- | --- | --- | --- | --- |
|  | Aandytaiji | BDJqigong | BDJqigongT | Control | Eytaijigame | LZJqigong | SEDJqigong | YFFqigong | Ytaiji | YtaijitDs | YWLBqigong |
| Aandytaiji | Aandytaiji | -2.38 (-6.78, 1.62) | -2.88 (-8.19, 1.92) | -4.28 (-8.26, -0.75) | -1.79 (-6.57, 2.82) | -1.86 (-7.4, 3.26) | 0.01 (-4.72, 4.19) | -2.24 (-7.74, 2.81) | -1.98 (-5.34, 1.41) | -3.94 (-8.61, 0.49) | -2.48 (-7.77, 2.32) |
| BDJqigong | 2.38 (-1.62, 6.78) | BDJqigong | -0.5 (-4.4, 3.34) | -1.9 (-3.8, -0.04) | 0.59 (-3.08, 4.45) | 0.53 (-3.69, 4.74) | 2.39 (-0.7, 5.34) | 0.14 (-4.05, 4.24) | 0.4 (-1.9, 3.11) | -1.57 (-5.09, 2.16) | -0.11 (-4.03, 3.77) |
| BDJqigongT | 2.88 (-1.92, 8.19) | 0.5 (-3.34, 4.4) | BDJqigongT | -1.4 (-4.77, 2.03) | 1.1 (-3.49, 5.91) | 1.05 (-4.02, 6.14) | 2.89 (-1.3, 6.99) | 0.65 (-4.38, 5.66) | 0.9 (-2.62, 4.95) | -1.07 (-5.54, 3.67) | 0.4 (-4.45, 5.22) |
| Control | 4.28 (0.75, 8.26)* | 1.9 (0.04, 3.8)* | 1.4 (-2.03, 4.77) | Control | 2.49 (-0.64, 5.86) | 2.43 (-1.32, 6.23) | 4.29 (1.87, 6.6)* | 2.04 (-1.67, 5.72) | 2.31 (0.87, 4.18)* | 0.33 (-2.63, 3.55) | 1.79 (-1.64, 5.22) |
| Eytaijigame | 1.79 (-2.82, 6.57) | -0.59 (-4.45, 3.08) | -1.1 (-5.91, 3.49) | -2.49 (-5.86, 0.64) | Eytaijigame | -0.07 (-5.16, 4.83) | 1.79 (-2.38, 5.64) | -0.46 (-5.54, 4.37) | -0.18 (-3.36, 3.2) | -2.17 (-6.48, 2.16) | -0.7 (-5.55, 3.9) |
| LZJqigong | 1.86 (-3.26, 7.4) | -0.53 (-4.74, 3.69) | -1.05 (-6.14, 4.02) | -2.43 (-6.23, 1.32) | 0.07 (-4.83, 5.16) | LZJqigong | 1.85 (-2.69, 6.26) | -0.4 (-5.68, 4.85) | -0.11 (-4.06, 4.21) | -2.1 (-6.87, 2.89) | -0.64 (-5.71, 4.45) |
| SEDJqigong | -0.01 (-4.19, 4.72) | -2.39 (-5.34, 0.7) | -2.89 (-6.99, 1.3) | -4.29 (-6.6, -1.87) | -1.79 (-5.64, 2.38) | -1.85 (-6.26, 2.69) | SEDJqigong | -2.24 (-6.58, 2.18) | -1.98 (-4.59, 1.19) | -3.96 (-7.68, 0.11) | -2.5 (-6.6, 1.73) |
| YFFqigong | 2.24 (-2.81, 7.74) | -0.14 (-4.24, 4.05) | -0.65 (-5.66, 4.38) | -2.04 (-5.72, 1.67) | 0.46 (-4.37, 5.54) | 0.4 (-4.85, 5.68) | 2.24 (-2.18, 6.58) | YFFqigong | 0.28 (-3.58, 4.56) | -1.7 (-6.4, 3.24) | -0.24 (-5.26, 4.81) |
| Ytaiji | 1.98 (-1.41, 5.34) | -0.4 (-3.11, 1.9) | -0.9 (-4.95, 2.62) | -2.31 (-4.18, -0.87) | 0.18 (-3.2, 3.36) | 0.11 (-4.21, 4.06) | 1.98 (-1.19, 4.59) | -0.28 (-4.56, 3.58) | Ytaiji | -1.97 (-5.17, 0.98) | -0.5 (-4.57, 3.04) |
| YtaijitDs | 3.94 (-0.49, 8.61) | 1.57 (-2.16, 5.09) | 1.07 (-3.67, 5.54) | -0.33 (-3.55, 2.63) | 2.17 (-2.16, 6.48) | 2.1 (-2.89, 6.87) | 3.96 (-0.11, 7.68) | 1.7 (-3.24, 6.4) | 1.97 (-0.98, 5.17) | YtaijitDs | 1.46 (-3.26, 5.96) |
| YWLBqigong | 2.48 (-2.32, 7.77) | 0.11 (-3.77, 4.03) | -0.4 (-5.22, 4.45) | -1.79 (-5.22, 1.64) | 0.7 (-3.9, 5.55) | 0.64 (-4.45, 5.71) | 2.5 (-1.73, 6.6) | 0.24 (-4.81, 5.26) | 0.5 (-3.04, 4.57) | -1.46 (-5.96, 3.26) | YWLBqigong |

1: BDJqigongandT: Baduanjin Qigong and Transcranial direct current stimulation therapy; 2 : BDJqigong:Baduanjin Qigong; 3:LZJqigong:Liuzijue Qigong;4:SEDJqigong: Twelve Duanjin Qigong; 5:YFFqigong: Yifei Fang Qigong; 6: YWLBqigong:Yijinjing Qigong, Wuqinxi Qigong, Liuzijue Qigong, Baduanjin Qigong; 7: EYtaijigame:It is body movement-controlled computer Yang stye Tai ji game; 8:Ytaiji:Yang stye of Tai ji; 9:AandYtaiji:Acupuncture combined with Yang stye of Tai ji; 10:YtaijitDcs:Yang stye of Tai ji combined with tDCS (transcranial Direct Current Stimulation); 11: RR: Risk Ratio; 12: CI: Confidence Interval.

Supplement Table 4. MMSE index intervention and league table of control group

| **RR(95%CI)** | | | | | | | |
| --- | --- | --- | --- | --- | --- | --- | --- |
|  | Control | Naoandtaiji | Qigong | QigongandT | Taiji | Taijiandqigong | Taijiball |
| Control | Control | 3.90 (-0.2, 8.07) | 1.15 (-0.81, 3.13) | 1.54 (-1.24, 4.32) | 1.42 (-0.32, 2.62) | 3.35 (-0.01, 6.72) | 8.74 (5.94, 11.53)* |
| Naoandtaiji | -3.90 (-8.07, 0.2) | Naoandtaiji | -2.75 (-7.31, 1.77) | -2.35 (-7.3, 2.55) | -2.53 (-7.08, 1.68) | -0.54 (-5.9, 4.77) | 4.84 (-0.1, 9.75) |
| Qigong | -1.15 (-3.13, 0.81) | 2.75 (-1.77, 7.31) | Qigong | 0.39 (-3.03, 3.8) | 0.26 (-2.47, 2.47) | 2.19 (-1.71, 6.1) | 7.58 (4.17, 11.01)* |
| QigongandT | -1.54 (-4.32, 1.24) | 2.35 (-2.55, 7.3) | -0.39 (-3.8, 3.03) | QigongandT | -0.12 (-3.55, 2.73) | 1.81 (-2.49, 6.13) | 7.2 (3.26, 11.11)* |
| Taiji | -1.42 (-2.62, 0.32) | 2.53 (-1.68, 7.08) | -0.26 (-2.47, 2.47) | 0.12 (-2.73, 3.55) | Taiji | 1.96 (-1.5, 5.83) | 7.32 (4.46, 10.76)* |
| Taijiandqigong | -3.35 (-6.72, 0.01) | 0.54 (-4.77, 5.9) | -2.19 (-6.1, 1.71) | -1.81 (-6.13, 2.49) | -1.96 (-5.83, 1.5) | Taijiandqigong | 5.39 (1.01, 9.74)* |
| Taijiball | -8.74 (-11.53, -5.94) | -4.84 (-9.75, 0.1) | -7.58 (-11.01, -4.17) | -7.20 (-11.11, -3.26) | -7.32 (-10.76, -4.46) | -5.39 (-9.74, -1.01) | Taijiball |

1: Naoandtaiji:Nao Ling Tang and Tai ji; 2:QigongandT:Qigong and Transcranial direct current stimulation therapy; 3: Etaijigame:Exergaming is body movement-controlled computer Tai ji game; 4:Taijiandqigong:Taiji combined qigong; 5: Taijiball:Tai ji soft ball exercise; 6: RR: Risk Ratio; 7: CI: Confidence Interval.

*: The data is statistically significant

Supplement Table 5 MMSEsubgroup index intervention and league table of control group

| **RR(95%CI)** | | | | | | | | |
| --- | --- | --- | --- | --- | --- | --- | --- | --- |
|  | BDJqigongT | Control | LZJqigong | Naoandytaiji | Taijiandqigong | Taijiball | Ytaiji | YWLBqigong |
| BDJqigongT | BDJqigongT | -1.54 (-5.46, 2.37) | -0.2 (-5.75, 5.38) | 2.37 (-3.91, 8.51) | 1.83 (-4.05, 7.67) | 7.21 (1.67, 12.74)* | -0.18 (-4.9, 3.87) | -0.55 (-6.11, 5.01) |
| Control | 1.54 (-2.37, 5.46) | Control | 1.34 (-2.61, 5.31) | 3.91 (-1.05, 8.77) | 3.36 (-1, 7.71) | 8.75 (4.81, 12.68)* | 1.35 (-0.91, 3) | 1 (-2.92, 4.93) |
| LZJqigong | 0.2 (-5.38, 5.75) | -1.34 (-5.31, 2.61) | LZJqigong | 2.57 (-3.72, 8.78) | 2.02 (-3.86, 7.86) | 7.41 (1.79, 12.97) | 0.01 (-4.71, 4.12) | -0.35 (-5.92, 5.21) |
| Naoandytaiji | -2.37 (-8.51, 3.91) | -3.91 (-8.77, 1.05) | -2.57 (-8.78, 3.72) | Naoandytaiji | -0.54 (-7.03, 6.02) | 4.84 (-1.34, 11.09) | -2.61 (-8.04, 2.51) | -2.91 (-9.06, 3.38) |
| Taijiandqigong | -1.83 (-7.67, 4.05) | -3.36 (-7.71, 1) | -2.02 (-7.86, 3.86) | 0.54 (-6.02, 7.03) | Taijiandqigong | 5.38 (-0.45, 11.25) | -2.05 (-7.06, 2.47) | -2.36 (-8.2, 3.51) |
| Taijiball | -7.21 (-12.74, -1.67) | -8.75 (-12.68, -4.81) | -7.41 (-12.97, -1.79) | -4.84 (-11.09, 1.34) | -5.38 (-11.25, 0.45) | Taijiball | -7.39 (-12.11, -3.31) | -7.74 (-13.28, -2.17) |
| Ytaiji | 0.18 (-3.87, 4.9) | -1.35 (-3, 0.91) | -0.01 (-4.12, 4.71) | 2.61 (-2.51, 8.04) | 2.05 (-2.47, 7.06) | 7.39 (3.31, 12.11)* | Ytaiji | -0.35 (-4.4, 4.35) |
| YWLBqigong | 0.55 (-5.01, 6.11) | -1 (-4.93, 2.92) | 0.35 (-5.21, 5.92) | 2.91 (-3.38, 9.06) | 2.36 (-3.51, 8.2) | 7.74 (2.17, 13.28)* | 0.35 (-4.35, 4.4) | YWLBqigong |

1: LZJqigong:Liuzijue Qigong; 2: NaoandYtaiji:Nao Ling Tang and Yang style of Tai ji; 3: Taijiandqigong:Taiji and Qigong is organized by combining Yang-style and Wu-style Tai ji withBa Duan Jin Qigong, Wu Qin Xi Qigong, and Liu Zi Jue Qigong; 4: Taijiball:Tai ji soft ball exercise; 5: YWLBqigong:Yijinjing Qigong, Wuqinxi Qigong, Liuzijue Qigong, Baduanjin Qigong; 6: Ytaiji:Yang stye of Tai ji; 7.BDJQigongandT:Baduanjin Qigong and Transcranial direct current stimulation therapy; 8: RR: Risk Ratio; 9: CI: Confidence Interval.

*:The data is statistically significant

Supplement Table 6 Meta-regression results of each intervention at different intervention times in the main outcome measures

| Outcome | Intervention | Course (RR(95%CI)) |
| --- | --- | --- |
| MOCA | Etaijigame | 1.86 (-0.92, 4.81) |
|  | Qigong | 2.35 (1.26, 3.36)* |
|  | QigongandT | 1.39 (-1.54, 4.33) |
|  | Taiji | 2.14 (0.79, 3.73)* |
|  | TaijitDCS | 0.24 (-2.36, 2.99) |
|  | Taiji.Aandtaiji | 1.98 (-0.92,4.85) |
| MOCA Subgroup | BDJqigong | 1.83 (-7.31, 8.73) |
|  | BDJqigongT | 2.11 (-16.60, 38.46) |
|  | Eytaijigame | 2.48 (-15.02, 29.26) |
|  | LZJqigong | 2.41 (-15.99, 17.45) |
|  | SEDJqigong | 3.97 (-26.56, 10.63) |
|  | YFFqigong | 2.31 (-25.69, 44.67) |
|  | Ytaiji | 2.38 (0.68, 4.48)* |
|  | YtaijitDs | 0.46 (-26.65, 59.72) |
|  | YWLBqigong | 1.70 (-7.10, 9.40) |
|  | Ytaiji.Aandytaiji | 3.05 (-18.80, 81.41) |
| MMSE | Naoandtaiji | 1.18 (-14.48, 48.56) |
|  | Qigong | 0.50 (-2.30, 5.40) |
|  | QigongandT | -5.42 (-35.27, 40.64) |
|  | Taiji | 0.66 (-0.87, 3.10) |
|  | Taijiandqigong | -0.81 (-95.15, 24.10) |
|  | Taijiball | 7.04 (-4.07, 29.94) |
| MMSE Subgroup | BDJqigongT | -25.89 (-94.75, 27.48) |
|  | LZJqigong | 0.24 (-4.11, 9.95) |
|  | Naoandytaiji | 0.55 (-15.59, 24.02) |
|  | Taijiandqigong | 0.15 (-22.10, 62.333) |
|  | Taijiball | 6.77(-3.11, 17.23) |
|  | Ytaiji | 0.52 (-1.51, 3.65) |
|  | EWLBqigong | -0.09 (-4.53, 6.33) |

1: RR: Risk Ratio; 2: CI: Confidence Interval.

*:The data is statistically significant

Supplement Table 6 Cochrane Collaboration's Specific Risk of Bias Evaluation Criteria

| Evaluation Criteria | Evaluation Result | Description of Evaluation Content |
| --- | --- | --- |
| 1. Random Allocation Method | Correct | - Random number table, computerized random number generation, drawing lots, or minimization method. |
|  | Incorrect | - Allocation based on date of birth, hospital or clinic number, etc., or any predictable method.  - Alternating allocation.  - Allocation based on doctor's judgment, patient choice, or experiment results. |
|  | Unclear | - The information provided is insufficient to judge whether the method is correct. |
| 2. Allocation Concealment | Adequate | - Centralized allocation, including telephone, internet, or pharmacy-controlled randomization.  - Sealed opaque envelopes.  - Coded allocations, not exposed to participants. |
|  | Inadequate | - Open random number tables or lists of allocation sequences.  - Non-sealed or non-opaque envelopes.  - Alternating allocation. |
|  | Unclear | - The information provided is insufficient to judge whether the allocation concealment is correct. |
| 3. Blinding | Correct | - Blinding was not necessary, but outcome assessments and measurements were unlikely to be affected (e.g., objective outcomes like death).  - Blinding was applied for both participants and key researchers, and the blinding was not compromised. |
|  | Incorrect | - Blinding was not applied or was inadequate, and the outcome assessment was likely affected. |
|  | Unclear | - The information provided is insufficient to judge whether the blinding was adequate. |
| 4. Completeness of Outcome Data | Complete | - All patients accounted for, including dropouts and missing data, reported with reasons provided.  - Intention-to-treat (ITT) analysis performed, if applicable.  - All outcome measures reported as planned, and data imputation methods were appropriate. |
|  | Incomplete | - Patient data missing or dropped out without reasons given.  - Data were selectively reported or not in line with initial study objectives. |
|  | Unclear | - The information provided is insufficient to judge whether the data was fully accounted for. |
| 5. Selective Reporting of Study Results | No Selective Reporting | - All outcomes specified in the protocol were reported.  - Data were presented for all main outcome indicators. |
|  | Selective Reporting | - One or more main outcomes were missing, and details were unclear in the reporting. |
|  | Unclear | -The information is incomplete and difficult to judge. |
| 6. Other Bias Sources | None | - No other sources of bias present. |
|  | Yes | -At least one major bias risk exists:  - Bias related to the study design used.  - Study terminated early (due to data reasons or legitimate termination reasons).  - Conflict of interest.  - Small sample size.  - Significant baseline imbalance.  - Claims of fraudulent behavior.  - Other issues. |
|  | Unclear | - Information is incomplete, difficult to judge whether major bias exists.  - Unclear whether the identified issue causes bias, and the reason or evidence is insufficient. |

Supplement Table 7. Detailed Explanation and Definition of Traditional Chinese Exercises and Their Combination with Medical Interventions

| Category | Intervention Method | Abbreviation | Description | Definition |
| --- | --- | --- | --- | --- |
| MOCA and MMEE group | Traditional Chinese Exercise | Qigong | Qi Gong | A traditional exercise that regulates energy ('Qi') through breathing, posture, and meditation (Yang et al., 2024). |
|  |  | Etaijigame | Exergaming-Based Tai Chi | A computer-based Tai Chi game controlled by body movements, integrating virtual reality technology (Yu et al., 2022). |
|  |  | Taiji | Tai Chi or Tai Ji | A traditional Chinese martial art combining body posture, breathing control, and meditation (Chen et al., 2023). |
|  |  | Taijiandqigong | Tai Chi and Qigong | A combination of Tai Chi and Qigong, achieving physical and mental balance through coordinated movements and breathing (Park et al., 2023). |
|  |  | Taijiball | Tai Ji soft ball exercise | Tai Ji soft ball is a Tai Ji equipment exercise using a racket similar to a badminton racket with a cloth surface and a leather ball filled with sand. It can be practiced solo or with a partner, incorporating the essence of Tai Ji (Wang et al., 2021). |
|  | Traditional Chinese Exercise Combined with Medical Techniques | QigongandT | Qigong and Transcranial Direct Current Stimulation | A practice combining Qigong with transcranial direct current stimulation (tDCS) to regulate brain functions through electrical stimulation (Jinping, 2022). |
|  |  | Aandtaiji | Acupuncture and Tai Chi | A therapy combining traditional acupuncture techniques with Tai Chi movements (Yu, 2021). |
|  |  | TaijitDcs | Tai Chi combined with Transcranial Direct Current Stimulation | A combination of Tai Chi with tDCS, using electrical currents to enhance brain and body coordination (Xu et al., 2023). |
|  |  | Naoandtaiji | Nao Ling Tang and Tai Ji | A therapy combining Nao Ling Tang herbal medicine with Tai Chi movements (Rizhen et al., 2013). |
| MOCA and MMSE subgroup | Traditional Chinese Exercise | BDJqigong | Baduanjin Qigong | A traditional Chinese exercise focusing on stretching and gentle movements to improve health and well-being (Xia et al., 2023). |
|  |  | LZJqigong | Liuzijue Qigong | A traditional Chinese breathing exercise, involving specific sounds and movements to enhance lung and organ function (Tuan and Juan, 2013). |
|  |  | SEDJqigong | Twelve Duanjin Qigong | A set of twelve traditional Chinese exercises aimed at improving physical flexibility, balance, and health (Hengjia et al., 2020). |
|  |  | YFFqigong | Yifei Fang Qigong | A traditional Chinese Qigong form that focuses on improving respiratory health and lung function (Min and Xiaodan, 2023). |
|  |  | YWLBqigong | Yijinjing Qigong, Wuqinxi Qigong, Liuzijue Qigong, Baduanjin Qigong | A combination of different traditional Chinese exercises aimed at improving overall health and balance (Jun and Zhongxing, 2018). |
|  |  | EYtaijigame | Body movement-controlled computer Yang style Tai Ji game | A computer-based game that uses body movements to control Yang-style Tai Ji practice through virtual reality (Yu et al., 2022). |
|  |  | Ytaiji | Yang style of Tai Ji | A traditional Chinese martial art focusing on slow, graceful movements to enhance physical and mental health (Li et al., 2014). |
|  |  | Taijiandqigong | Yang-style and Wu-style Tai Ji combined with Ba Duan Jin Qigong, Wu Qin Xi Qigong, and Liu Zi Jue Qigong | A practice combining multiple Tai Ji styles and Qigong exercises to promote physical and mental well-being (Park et al., 2023). |
|  |  | Taijiball | Tai Ji soft ball exercise | Tai Ji soft ball is a Tai Ji equipment exercise using a racket similar to a badminton racket with a cloth surface and a leather ball filled with sand. It can be practiced solo or with a partner, incorporating the essence of Tai Ji (Wang et al., 2021). |
|  | Traditional Chinese Exercise Combined with Medical Techniques | BDJqigongandT | Baduanjin Qigong and Transcranial direct current stimulation therapy | A combination of Baduanjin Qigong and transcranial direct current stimulation to enhance cognitive function and physical well-being (Jinping, 2022). |
|  |  | AandYtaiji | Acupuncture combined with Yang style of Tai Ji | A therapy combining acupuncture techniques with the Yang style of Tai Ji movements for improved health and balance (Yu, 2021). |
|  |  | YtaijitDcs | Yang style of Tai Ji combined with tDCS | A combination of the Yang style of Tai Ji and transcranial direct current stimulation to improve brain function and body coordination (Xu et al., 2023). |
|  |  | NaoandYtaiji | Nao Ling Tang and Yang style of Tai Ji | A therapy combining Nao Ling Tang herbal medicine with Yang style Tai Ji movements for enhanced physical and mental well-being (Rizhen et al., 2013). |

## References

Chen, Y., Qin, J., Tao, L., Liu, Z., Huang, J., Liu, W., Xu, Y., Tang, Q., Liu, Y., and Chen, Z. (2023). Effects of tai chi chuan on cognitive function in adults 60 years or older with type 2 diabetes and mild cognitive impairment in China: A randomized clinical trial. *JAMA network open* 6**,** e237004-e237004.

Hengjia, L., Yinxiumin, Qingnan, F., Jing, Y., and Jie, Z. (2020). Health qigong twelve Duan Jin impact study in patients with mild cognitive dysfunction *Chinese Journal of Preventive Medicine* 21**,** 965-968.

Jinping, F. (2022). Eight period of jin joint transcranial direct current stimulation in the treatment of mild cognitive dysfunction after stroke clinical studies *New Traditional Chinese Medicine* 54**,** 146-149.

Jun, C., and Zhongxing, Z. (2018). A Study on the Intervention Effects of Continuous Qigong Exercise on Mild Cognitive Impairment in the Elderly. *Journal of Baicheng Teachers College* 32**,** 59-63.

Li, F., Harmer, P., Liu, Y., and Chou, L.-S. (2014). Tai Ji Quan and global cognitive function in older adults with cognitive impairment: a pilot study. *Archives Of Gerontology And Geriatrics* 58**,** 434-439.

Min, Z., and Xiaodan, L. (2023). To explore the effect and mechanism of health qigong Yangfei decoction on cognitive function in patients with stable chronic obstructive pulmonary disease *Chinese Journal of Rehabilitation Medicine* 38**,** 904-910.

Park, M., Song, R., Ju, K., Shin, J.C., Seo, J., Fan, X., Gao, X., Ryu, A., and Li, Y. (2023). Effects of Tai Chi and Qigong on cognitive and physical functions in older adults: systematic review, meta-analysis, and meta-regression of randomized clinical trials. *BMC geriatrics* 23**,** 352.

Rizhen, L., Yunlin, L., Liangxin, W., Zhao, X.-H., and Pixiaohong (2013). Effect of Naoling Decoction combined with Tai Chi on rehabilitation of patients with Alzheimer's disease. *Modern magazine which combines traditional Chinese medicine with western medicine* 22**,** 693-694+760.

Tuan, Z.X., and Juan, Y. (2013). Health qigong six tactic. Study of effects of mild cognitive impairment rehabilitation of the age. *International Journal of Traditional Chinese Medicine* 35**,** 968-972.

Wang, Y.T., Goh, C.H., Liao, T., Dong, X.N., Duke, G., Alfred, D., Yang, Y., Xu, J., and Yu, S. (2021). Effects of wheelchair Tai Chi ball exercise on physical and mental health and functional abilities among elderly with physical disability. *Res Sports Med* 29**,** 289-302.

Xia, R., Wan, M., Lin, H., Ye, Y., Chen, S., and Zheng, G. (2023). Effects of mind–body exercise Baduanjin on cognition in community-dwelling older people with mild cognitive impairment: a randomized controlled trial. *Neuropsychological Rehabilitation* 33**,** 1368-1383.

Xu, Y., Zhu, J., Liu, H., Qiu, Z., Wu, M., Liu, J., Wu, J., Huang, J., Liu, Z., and Liu, W. (2023). Effects of Tai Chi combined with tDCS on cognitive function in patients with MCI: a randomized controlled trial. *Frontiers in public health* 11**,** 1199246.

Yang, H., Han, J., Wang, J., Duan, Y., and Jiang, J. (2024). Exploring the effects of combining health qigong and dance on working memory in middle-aged and elderly women: A preliminary investigation. *Exp Gerontol* 194**,** 112515.

Yu, A.P., Chin, E.C., Yu, D.J., Fong, D.Y., Cheng, C.P., Hu, X., Wei, G.X., and Siu, P.M. (2022). Tai Chi versus conventional exercise for improving cognitive function in older adults: a pilot randomized controlled trial. *Scientific reports* 12**,** 8868.

Yu, S.Z. (2021). *Clinical study of acupuncture and Tai Chi in the treatment of mild cognitive impairment caused by small cerebral vascular disease.* Master's degree.
