# Supplementary figures and images for: Effects of traditional Chinese exercises or their integration with medical treatments on cognitive impairment: a network meta-analysis based on randomized controlled trials
Source: Front Aging Neurosci. 2024 Nov 11;16:1475406. doi: 10.3389/fnagi.2024.1475406 (PMC11586772; doi:10.3389/fnagi.2024.1475406)

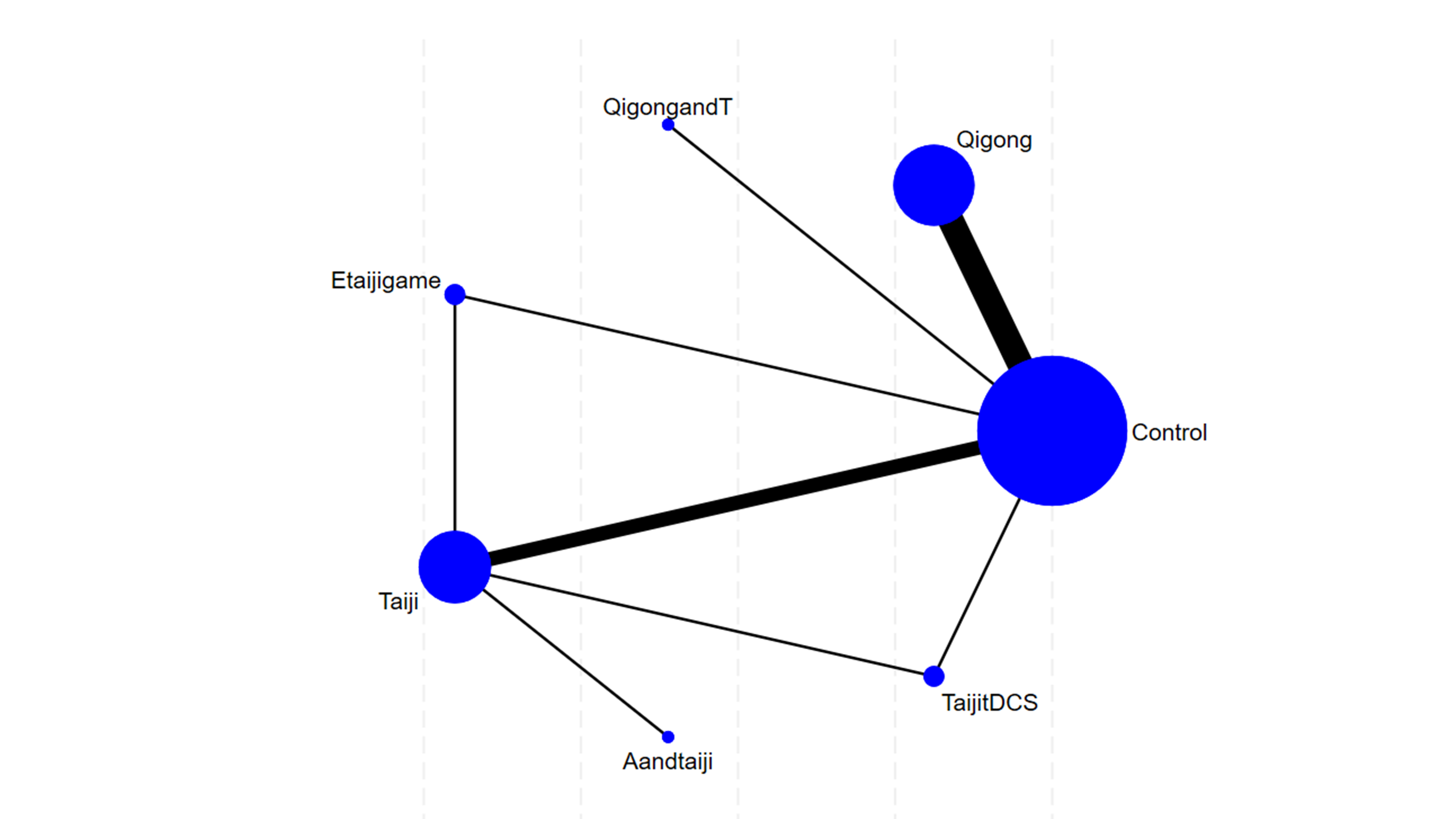

Supplement: Supplementary Figure 1 — MOCA index intervention and control group network relationship graph. 1: Qigong; 2: Qigong and tDCS; 3: Exergaming-based Tai Ji (Etaijigame); 4: Tai Ji; 5: Acupuncture and Tai Ji (Aandtaiji); 6: Tai Ji combined with tDCS (TaijitDcs). Method: This network plot visualizes comparisons between interventions and the control group. Node size represents the sample size, and edge thickness reflects the number of studies. Results: The control group and Tai Ji had the most data, represented by larger nodes and thicker lines. [file Image_1.tif]

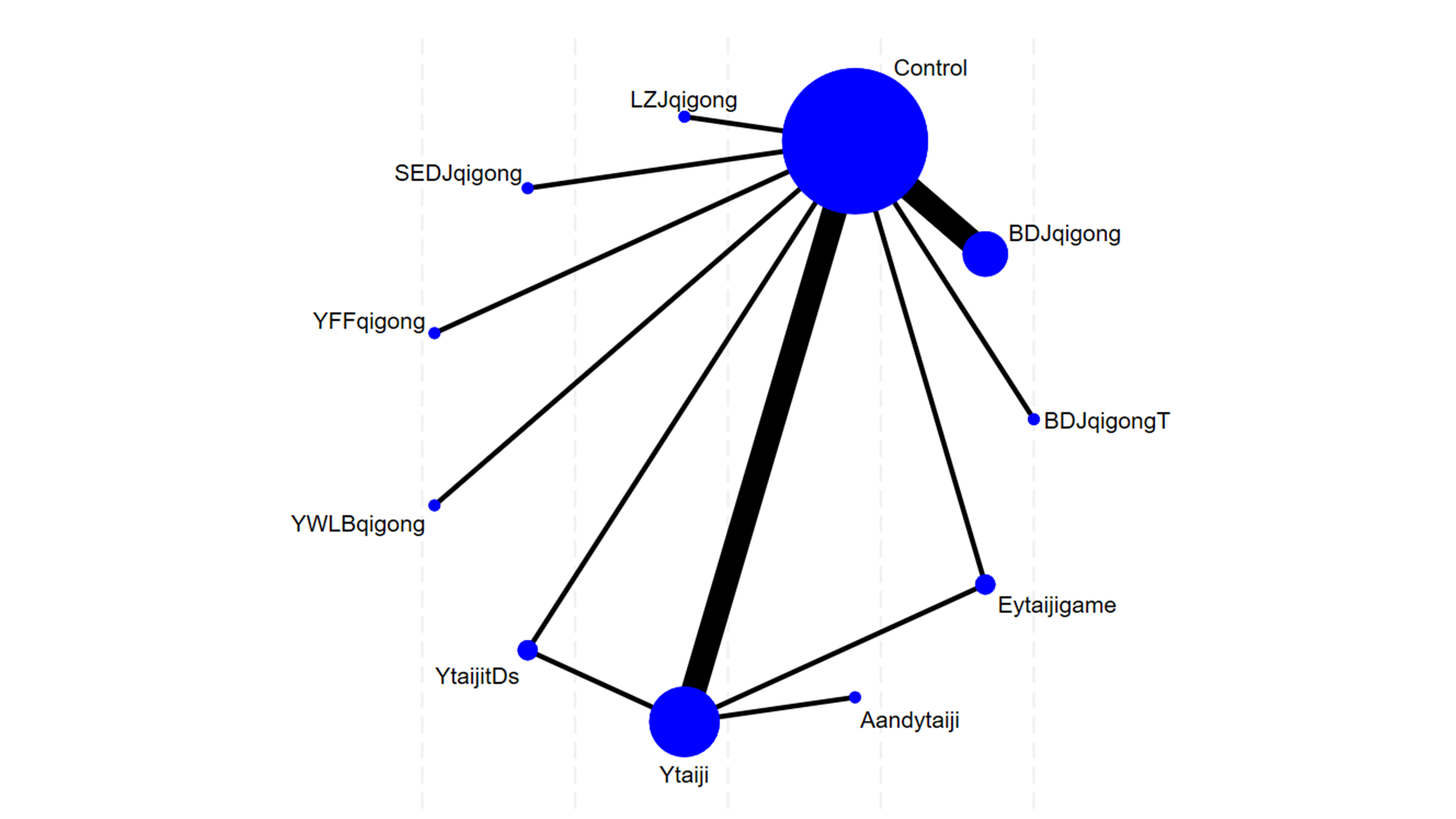

Supplement: Supplementary Figure 2 — MOCA subgroup intervention and control group network relationship graph. 1: Baduanjin Qigong + tDCS (BDJqigongandT); 2: Baduanjin Qigong (BDJqigong); 3: Liuzijue Qigong (LZJqigong); 4: Twelve Duanjin Qigong (SEDJqigong); 5: Yifei Fang Qigong (YFFqigong); 6: Yijinjing Qigong + Wuqinxi Qigong + Liuzijue Qigong + Baduanjin Qigong (YWLBqigong); 7: Yang-style Tai Ji game (EYtaijigame); 8: Yang-style Tai Ji (Ytaiji); 9: Acupuncture combined with Yang-style Tai Ji (AandYtaiji); 10: Yang-style Tai Ji combined with tDCS (YtaijitDcs). Method: A network plot displaying comparisons between subgroup interventions and the control group, with node size representing the sample size and edge thickness reflecting the number of comparisons. Results: Tai Ji and Qigong subgroups had the most comparisons with the control group. [file Image_2.tif]

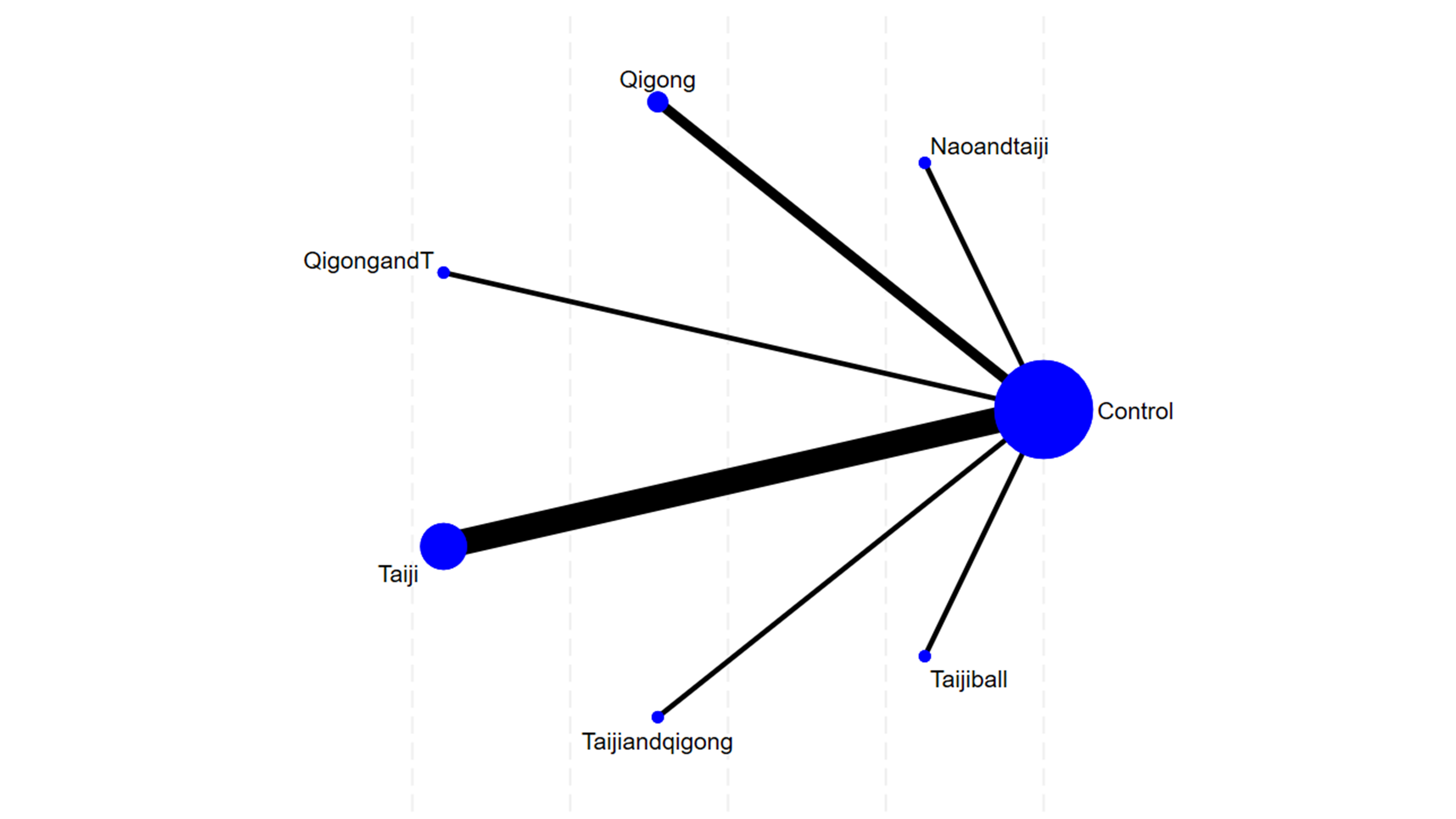

Supplement: Supplementary Figure 3 — MMSE index intervention and control group network relationship graph. 1: Nao Ling Tang and Tai Ji (Naoantaiji); 2: Qigong and tDCS (QigongandT); 3: Exergaming-based Tai Ji (Etaijigame); 4: Tai Ji and Qigong (combining Yang-style and Wu-style Tai Ji with Baduanjin, Wuqinxi, and Liuzijue Qigong); 5: Tai Ji soft ball exercise (Taijiball). Method: Nodes represent interventions, and edges show direct comparisons between interventions and the control group. Results: The control group was most frequently compared with interventions such as Tai Ji and Qigong, indicated by larger nodes and thicker lines. [file Image_3.tif]

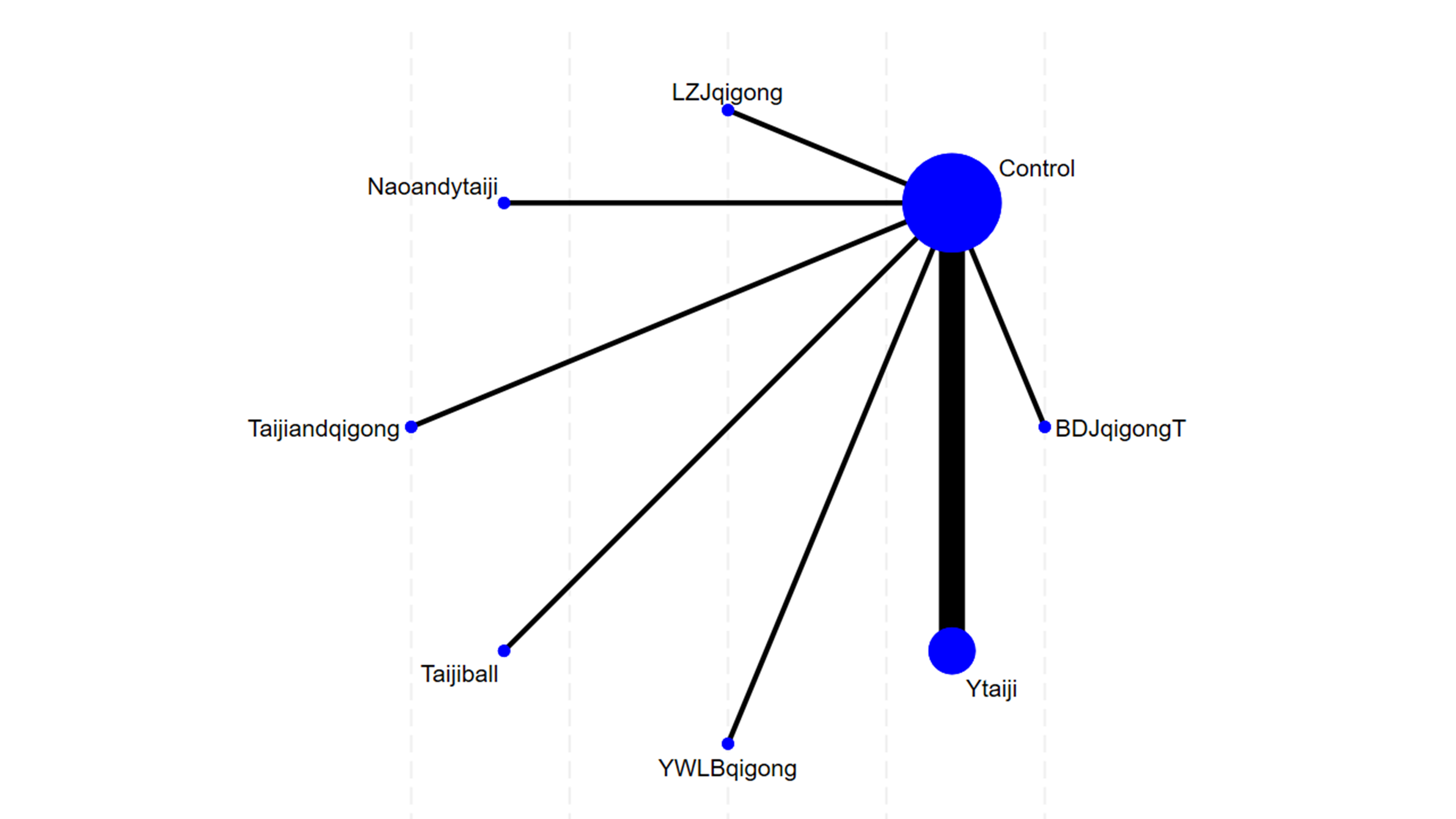

Supplement: Supplementary Figure 4 — MMSE subgroup intervention and control group network relationship graph. 1: Liuzijue Qigong (LZJqigong); 2: Nao Ling Tang and Yang-style Tai Ji (NaoanYtaiji); 3: Tai Ji and Qigong (combining Yang-style and Wu-style Tai Ji with Baduanjin, Wuqinxi, and Liuzijue Qigong); 4: Tai Ji soft ball exercise (Taijiball); 5: Yijinjing Qigong + Wuqinxi Qigong + Liuzijue Qigong + Baduanjin Qigong (YWLBqigong); 6: Yang-style Tai Ji (Ytaiji); 7: Baduanjin Qigong + tDCS (BDJQigongandT). Method: A network plot showing the relationship between various interventions and the control group. Results: The most frequent comparisons involved the control group and interventions such as Tai Ji and Qigong. [file Image_4.tif]
